# Silver Binding Dichotomy for 7‑Deazaadenine/Thymine: Preference for Watson–Crick Pairing over Homobase Interactions in DNA

**DOI:** 10.1021/acs.inorgchem.5c01762

**Published:** 2025-07-10

**Authors:** Carmen López-Chamorro, Antonio Pérez-Romero, Alicia Domínguez-Martín, Uroš Javornik, Oscar Palacios, Janez Plavec, Miguel A. Galindo

**Affiliations:** † Departamento de Química Inorgánica, 16741 Universidad de Granada, Avda. Fuentenueva s/n, 18071 Granada, Spain; ‡ Slovenian NMR Center, National Institute of Chemistry and Faculty of Chemistry and Chemical Technology, University of Ljubljana, SI-1000 Ljubljana, Slovenia; § Departament de Química, Facultat de Ciències, Universitat Autònoma de Barcelona, Cerdanyola del Vallès 08193, Spain

## Abstract

DNA strands modified with 7-deazaadenine (X) and 7-deazaguanine (Y) have shown promise in forming silver-DNA assemblies while maintaining canonical Watson–Crick base pairing, highlighting the compatibility of silver binding with standard DNA structures. However, critical questions remain regarding the binding preferences of Ag^I^ ions to sequences containing 7-deazapurine bases, particularly the prevalence of silver-modified Watson–Crick base pairs versus alternative homobase pair arrangements. To address this, we examined the binding of Ag^I^ to complementary X-T sequences, demonstrating a strong preference for canonical X-Ag^I^-T pairing over homoleptic X-Ag^I^-X or T-Ag^I^-T pairs. Additionally, we report the discovery of a novel metallized DNA duplex featuring continuous X-Ag^I^-X homobase pairs, whose structural analysis at the monomeric level, using model base 9-propyl-7-deazaadenine (pX) and Ag^I^ salts, reveals a unique silver-binding pattern through the Watson–Crick face. These findings not only advance our understanding of silver-mediated DNA architectures using 7-deazapurines but also provide a foundation for the rational design of sophisticated metal-DNA nanostructures with tailored properties, opening new avenues for the development of functional DNA-based materials.

## Graphical abstract

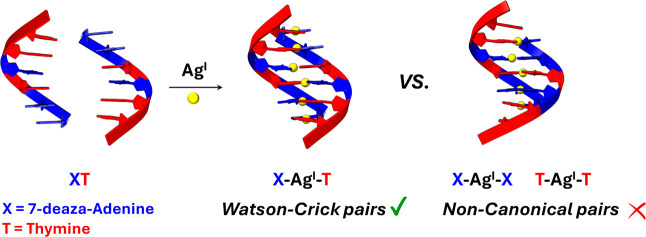

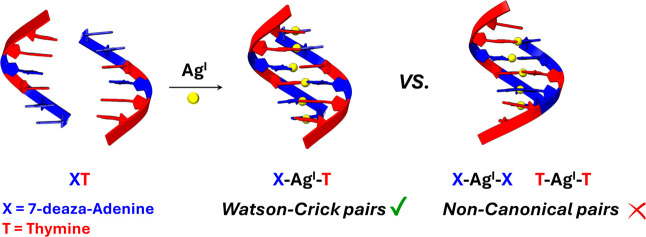

## Introduction

The development of predictable metal-DNA assemblies represents a transformative advancement in DNA-based nanotechnology, offering a versatile platform for designing novel functional materials with enhanced stability, tunable properties, and broad applicability.^1^ By strategically incorporating metal ions into DNA structures, researchers can endow these biomolecules with unique structural and functional characteristics, expanding their utility beyond natural biological roles.^2–5^ This integration enables the creation of sophisticated architectures where metal ions act as integral components, dictating the material’s properties and functionality. Silver ions, in particular, have demonstrated the ability to create silver-DNA systems conferring noninherent physicochemical properties to DNA, including higher stability,^6^ antibacterial activity,^7^ increased electrical conductivity,^8,9^ and the formation of fluorescent silver nanoclusters upon reduction.^10^ These capabilities have prompted growing interest in the rational design of silver–DNA constructs that incorporate non-native functionalities, thereby broadening the scope of DNA-based nanomaterials for applications in biosensing, drug delivery, molecular electronics, and beyond.

A particularly promising development is the use of silver–metalated base pairs, in which Ag^I^ ions are precisely positioned between bases, forming coordination bonds that replace hydrogen bonds, thereby offering a powerful strategy to modulate both DNA structure and behavior.^11–14^ In this context, modified nucleobases such as 7-deazaadenine (X) and 7-deazaguanine (Y) have emerged as unique tools for engineering metallo-DNA systems, maintaining canonical Watson–Crick base pairing.^15–17^ These purine analogues retain the essential base-pairing features of natural adenine and guanine while eliminating the N7 position, a common metal-binding site in canonical purine bases. By removal of the N7 site, 7-deazapurines redirect the Ag^I^-ion binding to the N1 position (Scheme 1). This structural alteration favors the formation of silver-modified base pairs that preserve the original Watson–Crick pairing framework, ensuring the DNA duplex maintains its native organization even in the presence of silver ions.^17^ Despite this progress, important questions remain regarding the binding preferences of silver ions in sequences containing 7-deazapurine bases. Previous studies have suggested the possible formation of silver-mediated X-Ag^I^-C and X-Ag^I^-G base pairs when initial mismatches such as X-C and X-G are present in the duplex.^18^ However, these findings do not account for potential sequence slippage events that may arise even in the absence of mismatches within sequences containing canonical base pairing. Such structural rearrangement has been observed in silver–metalated canonical DNA duplex^19^ and may similarly influence the formation of alternative metallo-base pairs in deaza-modified DNA (deazaDNA).

**Scheme 1:**
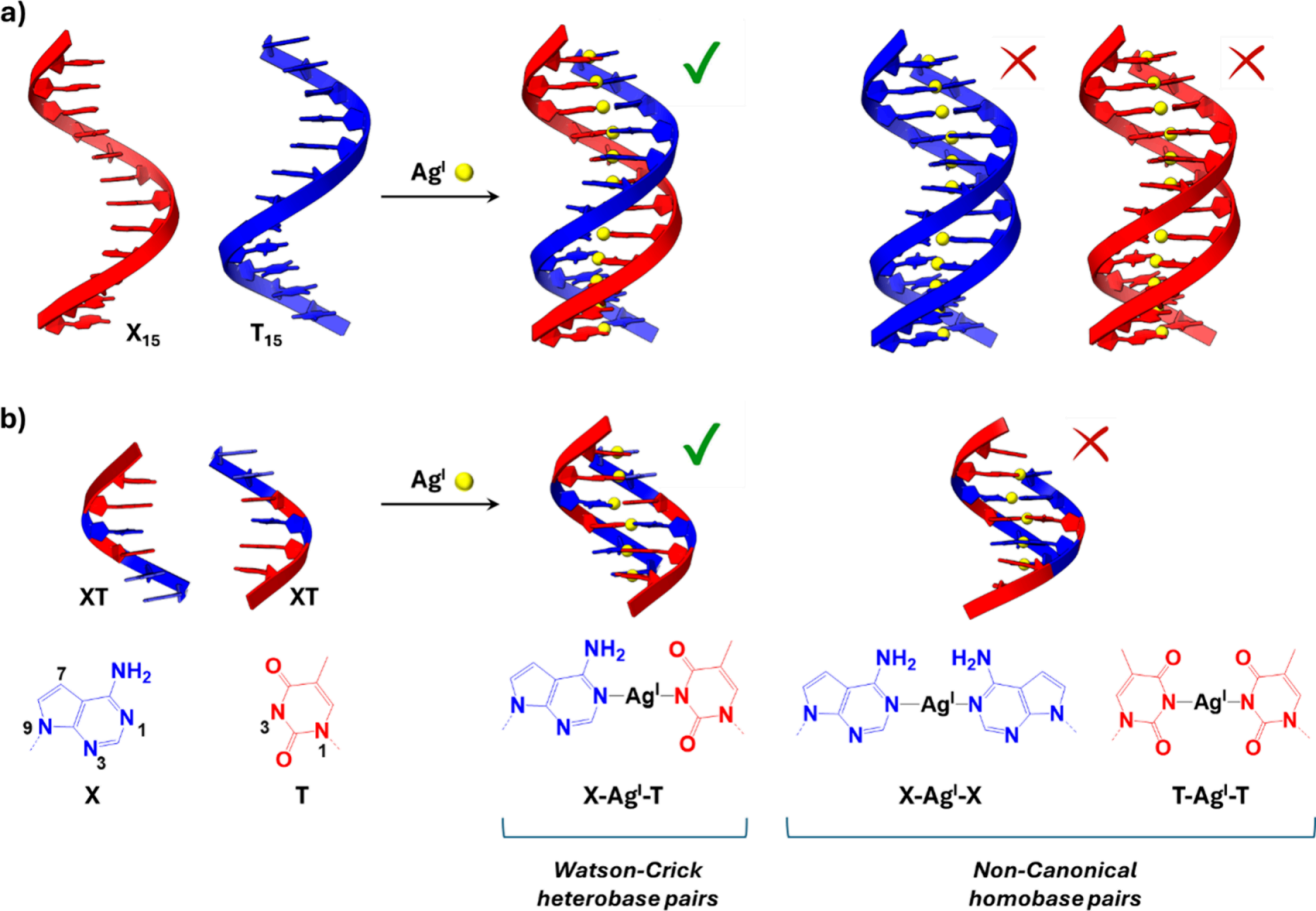
Illustration of the Reaction between Oligonucleotides Containing X (7-Deazaadenine) and T (Thymine) Bases with Ag^I^ Ions, Potentially Leading to the Formation of Duplexes Comprising X-Ag^I^-T Heterobase Pairs and X-Ag^I^-X or T-Ag^I^-T Homobase Pairs^a^

a These interactions may occur through (a) strand displacement or (b) strand slippage events.

In the present study, we aim to investigate the preferential formation of alternative silver–metalated base pairs involving 7-deazapurines in duplexes that are initially designed with canonical base pairing, without intentional mismatches (Scheme 1). This approach will provide new insights into the intrinsic base-pairing preferences and structural dynamics of metal-modified 7-deazaDNA systems.

In this context, herein, we first study the metalation of the X-T base pair. While prior studies have demonstrated that Ag^I^ ions effectively coordinate with X-T pairs to form stable silver-DNA duplexes,^16,17^ the potential formation of homobase pairs, such as X-Ag^I^-X or T-Ag^I^-T, remains poorly understood within these systems. These homobase pairs could disrupt the desired heterobase pair X-Ag^I^-T with a Watson–Crick arrangement, compromising the predictability and organization of metalized deazaDNA duplexes. Understanding these binding preferences is critical for the rational design of metal-deazaDNA systems with a controlled structure.

For our studies, we conducted a comparative study of Ag^I^ binding to homosequences (**X**_**15**_, **A**_**15**_, and **T**_**15**_) and heteroduplexes (**X**_**15**_ **·T**_**15**_ and **A**_**15**_ **·T**_**15**_), as well as a duplex **(XT)**_**6**_ formed using a self-complementary XT sequence (Table 1). The results obtained also revealed the formation of a new duplex featuring continuous X-Ag^I^-X base pairs. The structural features of this new silver-mediated base pair were also investigated by isolating and characterizing six Ag^I^ complexes using N9-propyl-7-deazaadenine (pX) as a model base.

**Table 1:** Sequences Employed in This Study

duplex	sequence^a^	sequence no.
**X**_**15**_ **·T**_**15**_	5′ - d(XXX XXX XXX XXX XXX) - 3′	**X**_**15**_
	3′ - d(TTT TTT TTT TTT TTT) - 5′	**T**_**15**_
		
**A**_**15**_ **·T**_**15**_	5′ - d(AAA AAA AAA AAA AAA) - 3′	**A**_**15**_
	3′ - d(TTT TTT TTT TTT TTT) - 5′	**T**_**15**_
		
**(XT)**_**6**_	5′ - d(XXT XTT) - 3′	**XT**
	3′ - d(TTX TXX) - 5′	**XT**

a X, 7-deazaadenine; A, adenine; *T,* thymine.

## Results and Discussion

### Selective Formation of Silver-Mediated X-Ag^I^-T Base Pairs in DNA Duplexes

The formation of hetero- and homobase pairs within DNA duplexes was initially investigated by using circular dichroism (CD) spectroscopy. In the absence of Ag^I^, an equimolar mixture of **X**_**15**_ and **T**_**15**_ exhibited a CD profile characteristic of a B-form duplex conformation, comparable to the related A_n_·T_n_ duplex.^20^ However, upon the incremental addition of Ag^I^ ions, significant changes in the Cotton effects were observed (Figure 1a). These spectral changes stabilized after the addition of 1–1.5 equiv of Ag^I^ per base pair (bp), suggesting a well-defined binding stoichiometry. The appearance of two isodichroic points at 220 and 238 nm further confirmed the transition between distinct structural states. In contrast, the CD spectra of the control mixture of **A**_**15**_ and **T**_**15**_ displayed a markedly different response upon Ag^I^ addition, indicating an alternative silver-induced structural reorganization (Figure S1). The observed spectral alterations and the final CD spectrum for the metalated duplex resemble those reported previously for duplex alternating X and T bases and suggesting the formation of silver–metalized DNA, likely involving X-Ag^I^-T base pairs.^16^ However, these CD spectra alone may not conclusively confirm the exclusive formation of heterobase pairs, as the observed spectra changes could also arise from the formation of two distinct duplexes formed by continuous homobase pairs X-Ag^I^-X and T-Ag^I^-T.

**Figure 1:**
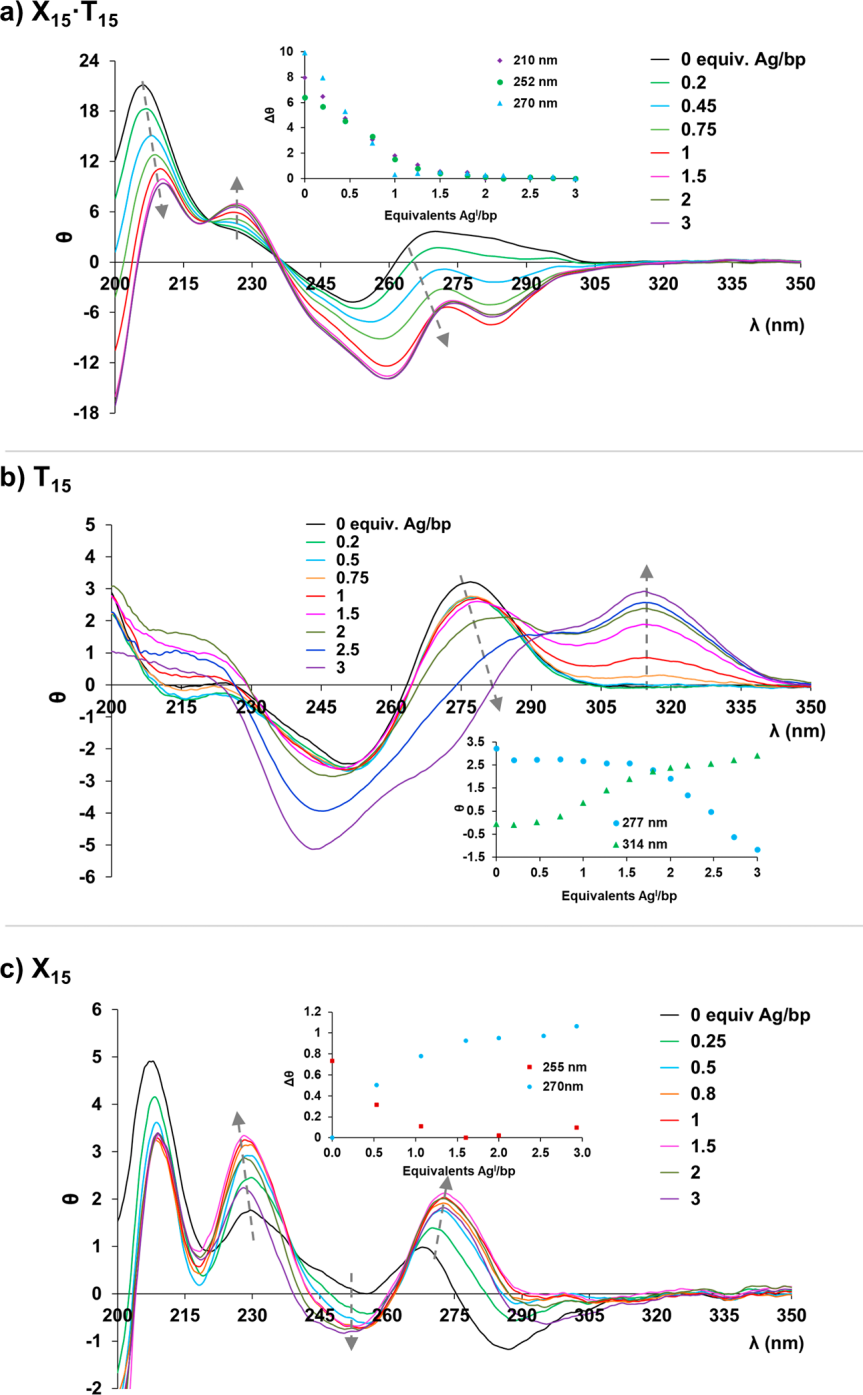
CD spectra of (a) duplex **X**_**15**_ **·T**_**15**_, (b) homosequence **T**_**15**_, and (c) homosequence **X**_**15**_, upon addition of different amounts of Ag^I^ ions per base pair (bp). Insets: Changes in the CD at the indicated wavelengths. Experimental conditions: 2 μM of a corresponding duplex, 100 mM NaClO_4_, and 5 mM MOPS buffer pH 6.8–7.

To assess the potential formation of homobase pairs, CD titration experiments were also performed on the individual homosequences **T**_**15**_ and **X**_**15**_, and the results were compared with the previous experiment. The interaction between Ag^I^ and **T**_**15**_ revealed different spectral features compared to duplex **X**_**15**_ **·T**_**15**_, providing insight into the binding behavior of Ag^I^. Initially, no significant changes were observed upon the addition of 1 equiv of Ag^I^ to **T**_**15**_ (Figure 1b). However, upon further Ag^I^ addition, notable alterations emerged, suggesting that an excess of Ag^I^ may be required to induce silver coordination to the thymine bases under the experimental conditions employed. The CD spectra also revealed the formation of intriguing species in the presence of an excess of Ag^I^ ions. A growing band at 320 nm indicated a manifest structural change, and a characteristic negative band at approximately 245 nm, typically associated with Ag^I^ binding to canonical DNA, was observed.

The interaction of Ag^I^ with thymine to form T-Ag^I^-T homobase pairs has been well-documented in both isolated complex^21^ and DNA duplex structures.^19^ Prior studies have also examined silver coordination in T_n_ homopolymers, including a T_16_ sequence similar to that studied here.^22^ However, our results showed a distinct conformational response, likely influenced by differences in experimental conditions. Unlike previous studies using Tris-acetate buffer, our experiments employed a nonchelating MOPS buffer, potentially leading to differential Ag^I^ binding behavior. Despite these methodological differences, both studies conclude that an excess of Ag^I^ is required to initiate conformational changes in thymidine-rich sequences, suggesting a threshold for effective silver coordination.

The CD titration of **X**_**15**_ with Ag^I^ also reveals a unique spectral profile different from that of **X**_**15**_ **·T**_**15**_ (Figure 1c). The free **X**_**15**_ strand exhibited two characteristic minima at 283 and 253 nm along with two maxima at 267 and 230 nm. Upon Ag^I^ addition, the intensity of the maxima increased, while the minimum at 253 nm decreased and the minimum at 283 nm became more pronounced. These major spectral shifts stabilized upon reaching approximately 1 equiv of Ag^I^ per base pair, indicating the formation of a well-defined silver–metallized structure. The observed binding stoichiometry strongly suggests the formation of X-Ag^I^-X homobase pairs, providing further insight into this novel silver-mediated base-pairing system. Further details on the structural characteristics of this newly formed silver-mediated homobase pair are described below.

By comparing the CD experiments described above, it becomes evident that each system exhibits a unique silver–metalized structure. Notably, the combined CD spectra of silver–metallized **X**_**15**_ **-Ag**_**15**_ and **T**_**15**_ **-Ag**_**15**_ do not reproduce the observed spectra of the silver–metallized **X**_**15**_ **·T**_**15**_ **-Ag**_**15**_ duplex. This finding provides evidence that oligonucleotides with sequences containing consecutive X and T base pairs preferentially form heterobase silver–metalized X-Ag^I^-T pairs rather than disrupting natural hydrogen-bonding base pairs to form homobase interactions. Furthermore, the binding preference was also assessed through a heating–cooling process, with CD spectra recorded before and after thermal cycling. The CD spectra of the silver–metallized **X**_**15**_ **·T**_**15**_ **-Ag**_**15**_ duplex remained unchanged after heating the sample to 90 °C and subsequently cooling it to 5 °C (Figure S2). This result implies that the formation of heterobase silver–metalized pairs is not merely a result of the initial assembly of the **X**_**15**_ **·T**_**15**_ duplex but is an inherent property of the system. The fact that the CD profile of **X**_**15**_ **·T**_**15**_ retains the same peak positions upon adding Ag^I^ (though with varying intensities) suggests that the Watson–Crick organization is likely maintained during the formation of the described silver–metallized pair. This interpretation is further supported by nuclear magnetic resonance (NMR) spectroscopy studies described below and by a recent study that resolved the solution structure of a DNA duplex comprising X-T and Y–C bases, demonstrating the formation of the corresponding silver-modified Waston–Crick bases.^17^

We next investigated the thermal stability of **X**_**15**_ **·T**_**15**_ in the absence and presence of Ag^I^ ions using temperature-variable UV spectroscopy. If Ag^I^ replaces Watson–Crick hydrogen bonding, duplex stabilization and a concomitant increase in melting temperature (*T*_m_) are expected. As shown in Figure 2, the **X**_**15**_ **·T**_**15**_ duplex exhibited a typical cooperative melting curve with a *T*_m_ of 13 °C in the absence of metal ions, consistent with standard hydrogen-bonded duplex formation.

**Figure 2:**
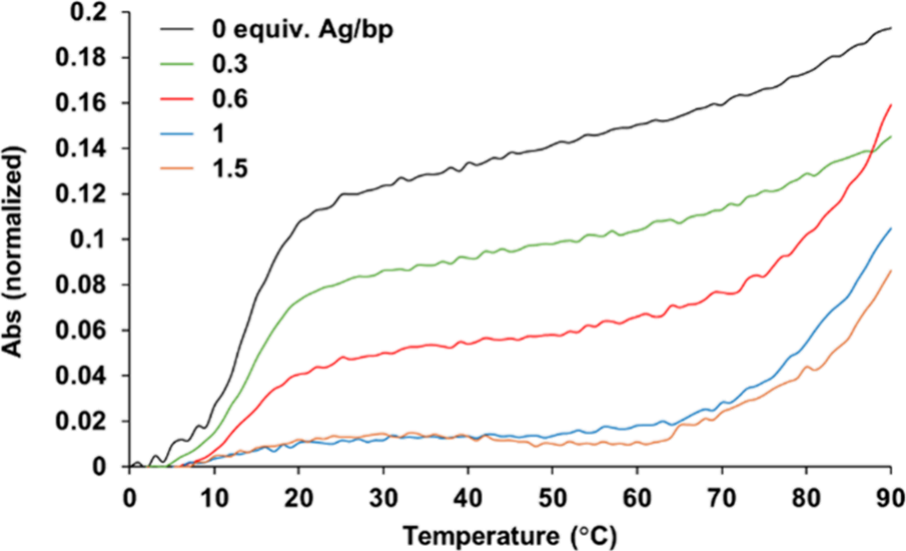
Normalized UV-melting curves recorded for duplex **X**_**15**_ **·T**_**15**_ in the absence and presence of different amounts of Ag^I^ ions per base pair (bp). Experimental conditions: 2 μM duplex, 100 mM NaClO_4_, and 5 mM MOPS buffer pH 6.8–7, AgNO_3_ 0 → 45 μM.

Upon Ag^I^ addition, the original melting curve gradually vanished and a new melting transition appeared above 70 °C. The upper limit of this transition was not observed because the experiment was limited to temperatures below 90 °C. Notably, rather than a gradual shift of the curves, the original transition disappeared entirely and a distinct new transition emerged at higher temperatures. This behavior agrees with the formation of X-Ag^I^-T base pairs and suggests a cooperative binding mechanism, where the duplex exists either in a metal-free or in a fully metalated state, without intermediate species. However, the precise nature of this metalation process remains an open question that is currently under investigation, extending beyond the scope of this study. The thermal stability of **X**_**15**_ was also examined in the presence of Ag^I^ ions. In the absence of metal ions, no cooperative melting transition was observed during heating, consistent with the lack of duplex formation (Figure S3). However, upon addition of Ag^I^, distinct cooperative transitions emerged and shifted to higher temperatures, indicating the formation of a silver-mediated duplex, consistent with the formation of X-Ag^I^-X base pairs, as also suggested by the CD experiments (vide supra).

Electrospray ionization mass spectrometry (ESI-MS, negative mode) was employed to further validate the formation of the **X**_**15**_ **·T**_**15**_ **-Ag**_**15**_ complex with expected metallized pairs. The deconvoluted mass spectrum of **X**_**15**_ **·T**_**15**_ in the presence of Ag^I^ ions displayed prominent peaks corresponding to the binding of 14 and 15 Ag^I^ ions (10,615.8 and 10,723.0 g·mol^–1^, respectively), providing direct evidence for the formation of well-defined silver-modified DNA duplexes (Figure 3 and Table S1). These results unequivocally confirm the incorporation of silver ions into the **X**_**15**_ **·T**_**15**_ structure, reinforcing the findings presented in this study.

**Figure 3:**
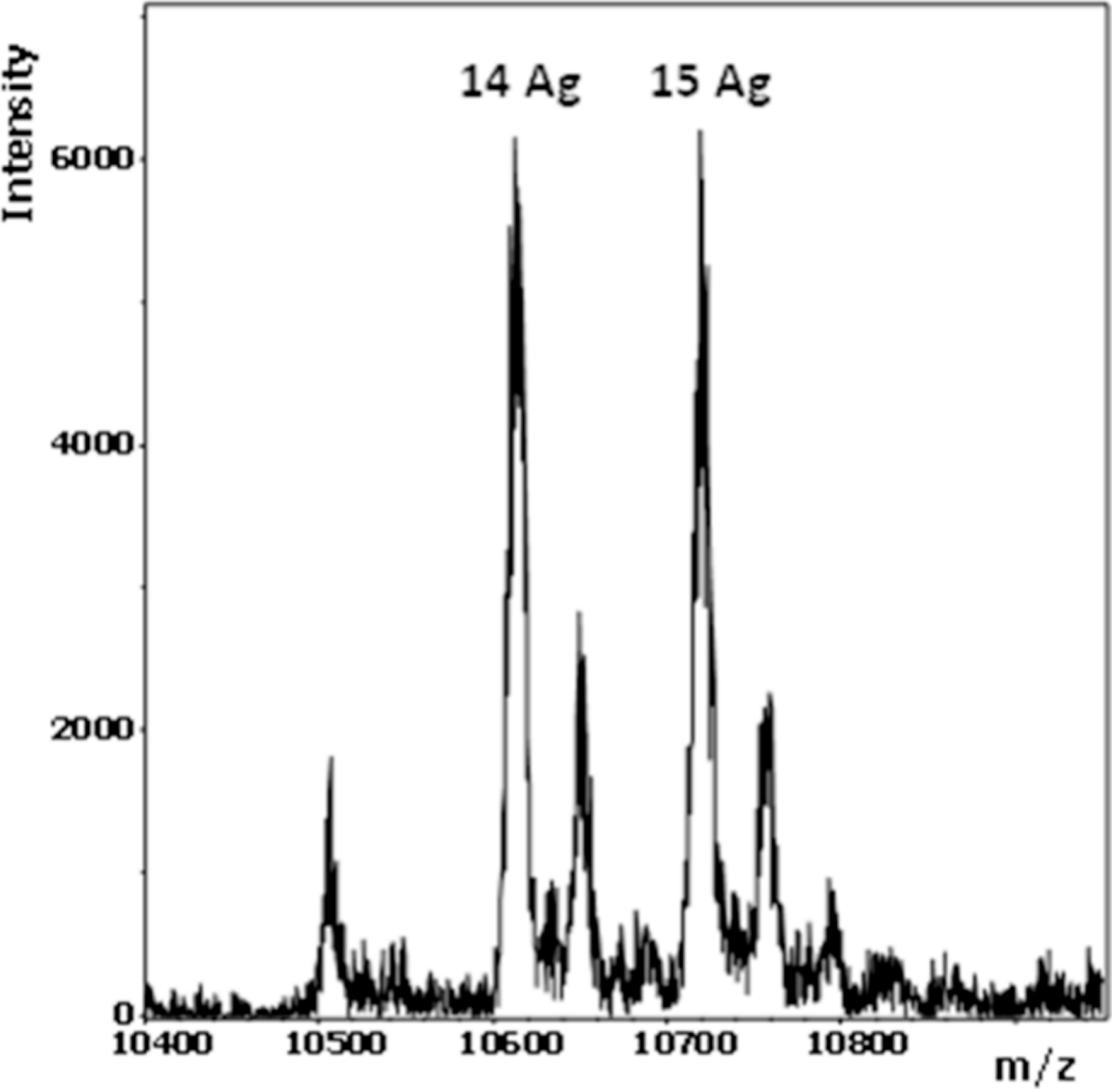
Deconvoluted ESI-MS spectrum of **X**_**15**_ **·T**_**15**_ in the presence of AgNO_3_. Conditions: 25 μM duplex, 100 mM NaClO_4_, and 5 mM MOPS buffer pH 6.8 and 825 μM AgNO_3_ (excess of 2.2 equiv Ag/bp).

To gain deeper insights into the preferential formation of the X-Ag^I^-T heterobase pair, we titrated a self-complementary hexanucleotide sequence alternating X and T bases (forming duplex **(XT)**_**6**_) with Ag^I^ and recorded ^1^H NMR spectra at 5 °C to monitor the process (Figures 4 and S4). If alternative silver-mediated homobase pairs were to form, sequence slipping would occur, leading to the formation of noncanonical base pairing and possible oligomerized silver-DNA species. This would result in the broadening or loss of NMR signals as the molecular weight increases, a phenomenon previously observed upon adding Ag^I^ to canonical DNA.^19,23^ The **XT** oligonucleotide initially remained in a single stranded form due to the short length of the sequence and the inherent lower stability of X-T base pairs. Upon the initial additions of Ag^I^, the ^1^H NMR signals began to shift, and additional signals were observed, indicating an equilibrium in which Ag^I^ ions interact with multiple binding sites along the oligonucleotide, with intermediate to slow exchange between the formed species. With continuing additions of Ag^I^, the signals gradually converged into a single set (between 1 and 2 equiv Ag^I^/bp), indicating the formation of distinct silver–metallized species. The signals corresponding to residues in the outer base pairs (X1, X2, T5, and T6) continue shifting, suggesting ongoing exchange with bulk Ag^I^, whereas those associated with inner base pairs (T3 and X4) remained stable, indicating a more protected environment. This behavior is analogous to hydrogen bonding in a canonical DNA duplex, implying that Ag^I^ ions are shielded within an **(XT)**_**6**_ duplex structure. Additionally, the upfield shift observed in the methyl proton signals of thymine in the metalated structure suggests enhanced aromatic base stacking interactions, as those seen during nucleic acid folding. The translational diffusion coefficient of the silver complex measured by DOSY NMR was lower than that of the free oligonucleotide form (1.49 ± 0.05 compared to 1.97 ± 0.03 × 10^–10^ m^2^·s^–1^, respectively, measured at 25 °C), suggesting an increase in the hydrodynamic radius, consistent with a transition from a single- to double-stranded **(XT)**_**6**_ **-Ag** structure. These observations strongly indicate that Ag^I^ binds between X and T bases in a manner analogous to that of the canonical AT base pair, leading to the formation of a discrete double-helical structure.

**Figure 4:**
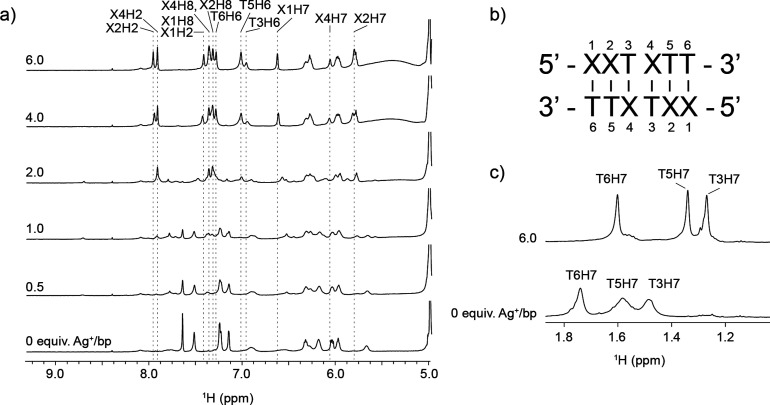
(a) Anomeric-aromatic region of the ^1^H NMR spectra of **(XT)**_**6**_ at different points along the titration with AgNO_3_ at 5 °C. Final positions of the aromatic proton signals are indicated with dashed lines, and assignments are displayed above the spectra. (b) Sequence of the **(XT)**_**6**_ oligonucleotide with residue numbering. (c) Methyl region of the ^1^H NMR spectra of **(XT)**_**6**_ at the start and end points of titration with AgNO_3_ at 5 °C; assignments of methyl proton signals are shown above the peaks.

Therefore, the data presented herein consistently demonstrate a preference for X-Ag^I^-T base pairs over homobase pair alternatives. Notably, previous mass spectrometry studies have also indicated a potential preference for A-Ag^I^-T heterobase pairs in canonical A and T sequences.^24^

### Silver-Mediated X-Ag^I^-X Base Pairs: Binding Interactions and Molecular Structure

As previously noted, the CD spectroscopy analysis of **X**_**15**_ binding with Ag^I^ ions revealed a system that reaches saturation at a stoichiometry of approximately one Ag^I^ ion per base pair (Figure 1c), suggesting the formation of a duplex containing consecutive X-Ag^I^-X homobase pairs. The formation of this silver–metalated duplex was further supported by UV-melting curve studies (Figure S3). This novel metallized DNA architecture has not been reported before. To model the binding interactions, we conducted a series of experiments using pX as a representative model nucleobase. The propyl group substitutes for the nucleobase sugar moiety, preventing metal coordination at the N9 position and thereby better replicating the metal-nucleobase binding modes in DNA.

Our initial approach involved solution studies via ^1^H NMR titrations, where pX was incrementally titrated with Ag^I^ ions (Figure 5). The NMR spectral analysis unveiled a noticeable downshift in the proton signals of pX, particularly pronounced in the case of the amino signal. These changes can be attributed to the binding of silver ions to the base. Notably, these shifts stabilized upon addition of 1–2 equiv of Ag^I^ per ligand, suggesting that a slight excess of silver is required to achieve full saturation of pX binding sites. Beyond this stoichiometry, further additions of Ag^I^ did not induce any spectral changes.

**Figure 5:**
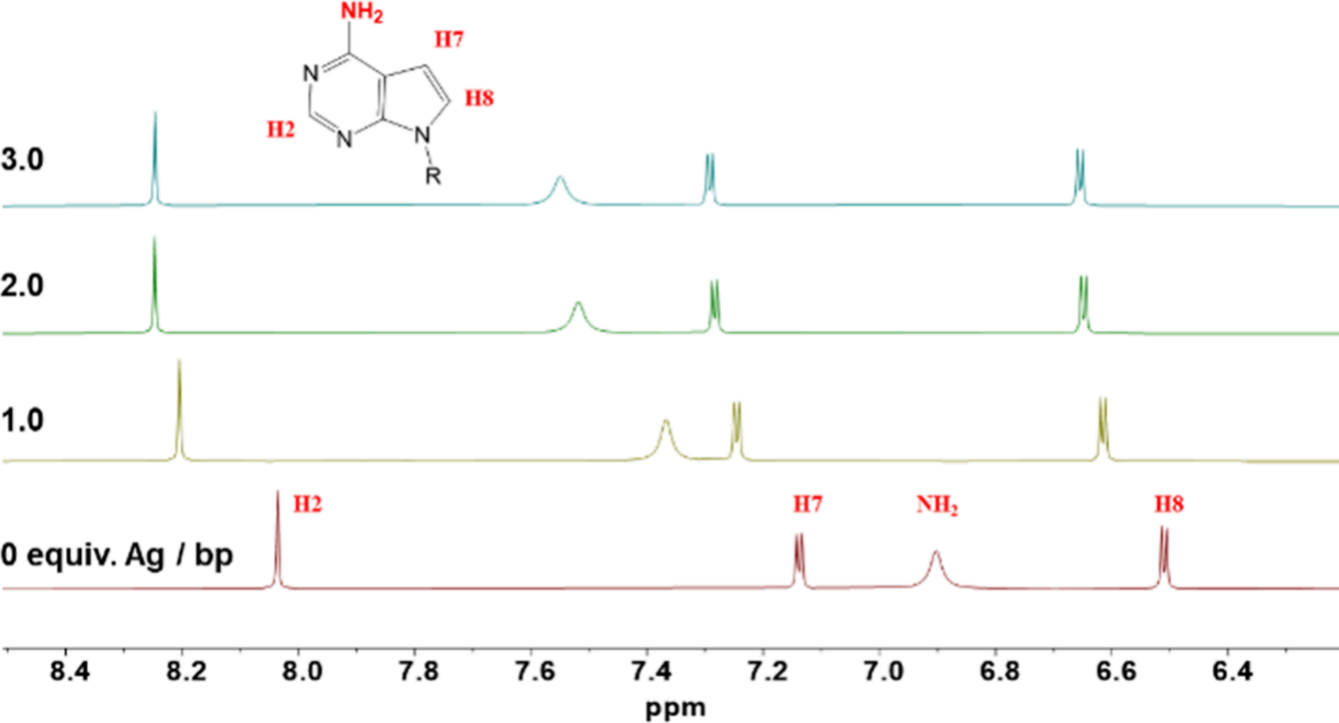
^1^H NMR (DMSO-d_6_) spectra of the aromatic region obtained during the titration experiment, where a constant amount of pX was mixed with increasing amounts of Ag^I^ ions. R, propyl chain; bp, base pair.

Following the solution studies, we explored the structural details of this new silver-mediated base pair through X-ray diffraction studies of silver complexes derived from pX. Several coordination compounds were synthesized and crystallized using different Ag^I^ salts, leading to the isolation of the following complexes: [Ag­(*N1*-pX)_2_]·nH_2_O (Z) (Z = ClO_4_, **1**; NO_3_, **2**; BF_4_, **3**; CF_3_SO_3_, **4**), [Ag_4_(*N1,N3-*pX)_4_(ClO_4_)_2_]­(ClO_4_)_2_ (**5**), and [Ag­(*N1*-pX)_3_]­(Cl) (**6**). Their molecular structures were elucidated by using single-crystal X-ray diffraction (Table S2).

The molecular structures of complexes **1**, **2**, **3,** and **4** feature a central Ag^I^ ion coordinated with two pX ligands, forming a distinctive silver-mediated homobase pair. In each complex, the Ag^I^ ion adopts a linear geometry and coordinates to pX bases via the N1 atom. While all cases exhibit a nearly coplanar configuration, notable differences are observed. Complex **1** crystallizes in the *P*2_1_/*n* space group, while **2** and **3** crystallize in the 2_1_/*c* space group. In these **1**–**3** complexes, the base pairs adopt a transoid arrangement, positioning the amino groups at a maximal distance from one another to minimize steric repulsion (Figure 6a). This arrangement optimally mitigates steric repulsion between amino groups. This spatial orientation also causes the propyl groups at the N9 position, which are analogous to the sugar units in nucleotides, to adopt a transoid configuration, resembling the base pairing observed in parallel DNA duplexes. The Ag–N1­(pX) distances for complexes **1**–**3** are consistently found within the range 2.125–2.152 Å, while the N–Ag–N angle varies between 171.63 and 173.36°. Additionally, the separation between N9 atoms of the two bases falls within the 12.209–12.288 Å range.

**Figure 6:**
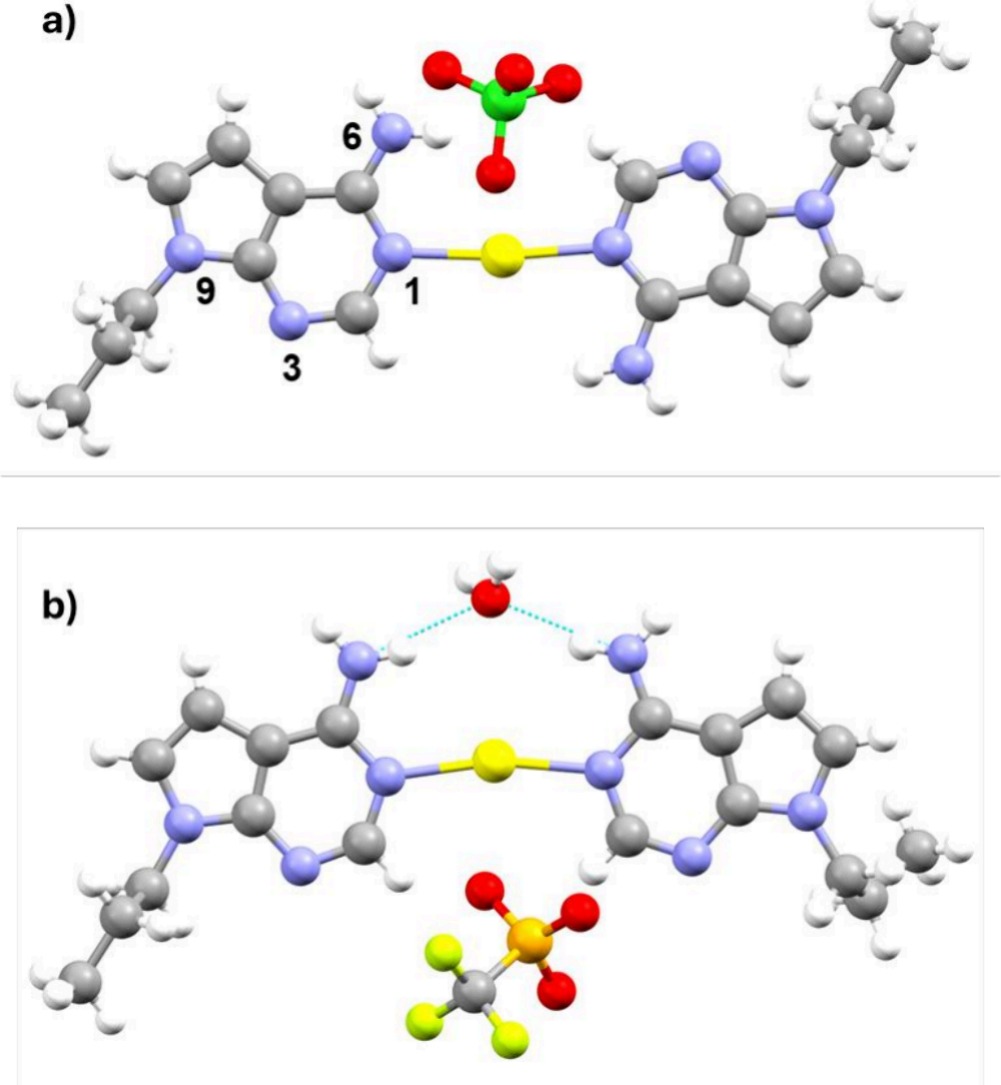
(a) Molecular structure of [Ag­(*N1*-pX)_2_]­(ClO_4_)·H_2_O (**1**) showing a base parallel arrangement as it would occur in a DNA duplex (water molecule omitted for clarity). (b) Molecular structure of [Ag­(*N1*-pX)_2_]_2_(CF_3_SO_3_)_2_·5H_2_O (**4**) displaying a base antiparallel arrangement, as it would occur in a DNA duplex (only one metal-mediated base pair with the corresponding counteranion and one water molecule are represented for the sake of clarity). Color code: carbon, gray; nitrogen, blue; oxygen, red; silver, yellow; chlorine, green; sulfur, orange; fluoride, light green; hydrogen, white.

In contrast, complex **4** crystallizes in the *P*-1 space group, and its molecular structure reveals a cisoid orientation of the base components, akin to the configuration found in antiparallel DNA structures (Figure 6b). This distinct conformation arises from the formation of hydrogen bonds between the amino groups and a shared water molecule, which causes the amino groups to face one another. In this complex, the Ag–N bond lengths range from 2.140 and 2.148 Å. However, the N–Ag–N angle is significantly reduced to 164.85°, and the distance between N9 atoms decreases to 12.073 Å. These changes result from the cisoid arrangement of the bases, which brings the amino groups closer together, a configuration stabilized by hydrogen bonding with the shared water molecule.

Complexes **1**–**3** exhibit notable similarities in their supramolecular structure. In these complexes, pairs of Ag-pX metallo-base pairs form zigzag ribbons that extend along the *b* axis, stabilized by reciprocal (exocyclic amino) N6–H···N3 hydrogen bonds, leading to a final motif that resembles a square metallocyclic. The corresponding counteranions (ClO_4_ (**1**), NO_3_ (**2**), and BF_4_ (**3**)) are placed at the center and around such a square, connecting the ribbons and the water molecules and thereby creating a hydrogen-bonded 3D network. Noteworthy, relevant π,π-stacking interactions are observed only in the crystal structure of **3**, where adjacent pX rings exhibit a distance of 3.87 Å between the six-membered rings, with angles α = 0° and β = γ = 26.8°. Other weak noncovalent interactions such as Ag···π and C–H···π are also present (see “Weak covalent interactions report”, Supporting Information). Crystal packing forces contribute to the geometric isomerism observed in compounds **1**–**4**, suggesting distinct intra- and intermolecular interactions. In complex **4**, the metallosquare motif is absent. Instead, pairs of base pairs are connected by CF_3_SO_3_ and H_2_O ligands, resulting in a complex hydrogen-bonded staircase arrangement. Antiparallel π,π-stacking and Agπ and C–Hπ interactions among neighboring purine moieties complete the 3D network of compound **4** (see “Weak covalent interactions report”, Supporting Information).

Complex **5** crystallizes in the *P*-1 space group. The asymmetric unit consists of a square metallocyclic structure formed by four Ag^I^ ions, four pX ligands, and four perchlorate ions, two of which are coordinated to Ag^I^ and located at opposite locations of the square (Figure 7).

**Figure 7:**
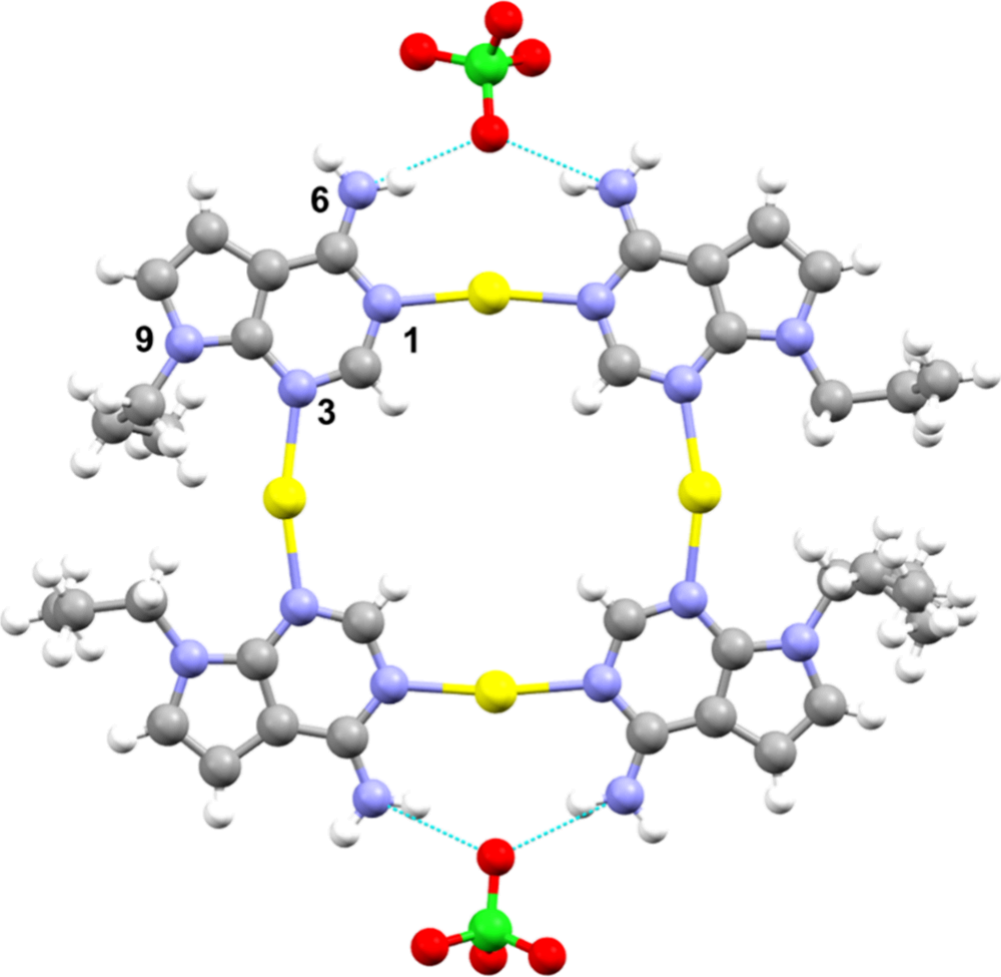
Molecular structure of [Ag_4_(*N1,N3*-pX)_4_(ClO_4_)_2_]­(ClO_4_)_2_ (**5**). Two perchlorate anions have been omitted for the sake of clarity. Color code: carbon, gray; nitrogen, blue; oxygen, red; silver, yellow; chlorine, green; hydrogen, white.

Each Ag^I^ is coordinated with two pX bases, where each base acts as a bidentate ligand via the N1 and N3 atoms. The metallocycle can be visualized as two metal-mediated base pairs, resembling the cisoid organization found in complex **4** (Figure 6b), linked via Ag^I^ ions that coordinate at their N3 atoms. In this organization, the amino groups are exposed to the exterior of the metallocycle. Two Ag^I^ ion ions adopt a linear geometry and coordinate to pX via N1 [Ag1–N1A 2.143, Ag1–N1D 2.146, Ag3–N1C 2.133, Ag3–N1B 2.138 Å]. The other two Ag^I^ ions adopt a T-shaped geometry and coordinate to the pX via N3 atoms [Ag2–N3A 2.183, Ag2–N3B 2.186, Ag4–N3C 2.205, and Ag4–N3D 2.202] and to one perchlorate anion, respectively (Ag2–O 2.673 Å; Ag4–O 2.705 Å). These perchlorates are located on opposite sides of the plane defined by the metallocycle. This arrangement leaves the exocyclic amino groups and the N9-propyl chain in a cisoid conformation relative to the base pair. The distances between the facing amino group are 5.221 Å (N6A···N6D) and 5.057 Å (N6B···N6C). These distances are smaller than those observed in **4** (also in cisoid conformation) since no water-mediated hydrogen-bonding interactions are present. The distances between opposite Ag^I^ ions are 7.972 (Ag1···Ag3) and 8.309 Å (Ag2···Ag4). The distances between N9 atoms in each base pair are 12.212 and 12.225 Å, respectively. Similar cisoid Pt­(II)-metallasquares involving 9-methylpurine and 9-methylhypoxanthine ligands were previously described by Lippert et al.^25,26^ As expected, these motifs always involved the N7-atom within the N1,N7 bridging mode. On the contrary, the absence of the N7-atom in pX drives coordination through the sole available heterocyclic N-donor of the purine moiety, leading to the N1,N3 bridging mode instead. This mode was previously reported only once in a copper­(II) complex for the neutral 7-deazaadenine ligand.^27^

The two noncoordinating perchlorate anions connect adjacent metallocycles bridging two exocyclic amino groups by formation of hydrogen bonds [N21···O12 2.867 Å and N4···O12 2.840 Å and N10···O5 Å 2.995 and N12···O5 2.901 Å], leading to chains that extend along the *c* axis. Neighboring chains interact via H-bonding involving the coordinated perchlorate ions and strong π,π-stacking interactions [d_c‑c_ 3.580 Å, α = 5°, β = 15.7°, γ = 12.5° and d_c–c_ 3.557 Å, α = 4°, β = 16.5°, γ = 13.1°]. Additional weaker noncovalent interactions further stabilize the 3D structure of the crystal, including H-bonds, π,π-stacking, and Ag···π interactions (see “Weak covalent interactions report”, Supporting Information).

Complex **6** was unexpectedly obtained by the addition of AgNO_3_ to a solution containing twice the equimolar amount of the pX reaction. The solution was filtered and left to crystallize, affording a minimal number of crystals suitable for X-ray diffraction. The analysis revealed the formation of complex **6** (Figure 8). The presence of the chloride anion must be due to the presence of impurities in the reaction mixture that could not be detected. This complex crystallizes in the monoclinic 21:*c* space group. The asymmetric unit consists of three pX ligands bonded to a Ag^I^ center via their corresponding N1 atoms and an apical coordinated chloride atom. The described coordination bonds are reinforced by their corresponding intramolecular hydrogen-bonding interactions, involving one H-atom from the exocyclic amino groups of each purine moiety as the H-donor and the chloride atom as the triple H-acceptor [average values N6···Cl 3.192 Å, 161°). Adjacent molecular complexes connect to each other by hydrogen bonds involving the pX ligands [N6–H···N3] in a straight fashion, leading to chains that extend along the *c* axis. C–H···π and N–H···π interactions among chains finally lead to the 3D architecture of the crystal, whose shape resembles the Celtic symbol Triskelion (Figure 8, right).

**Figure 8:**
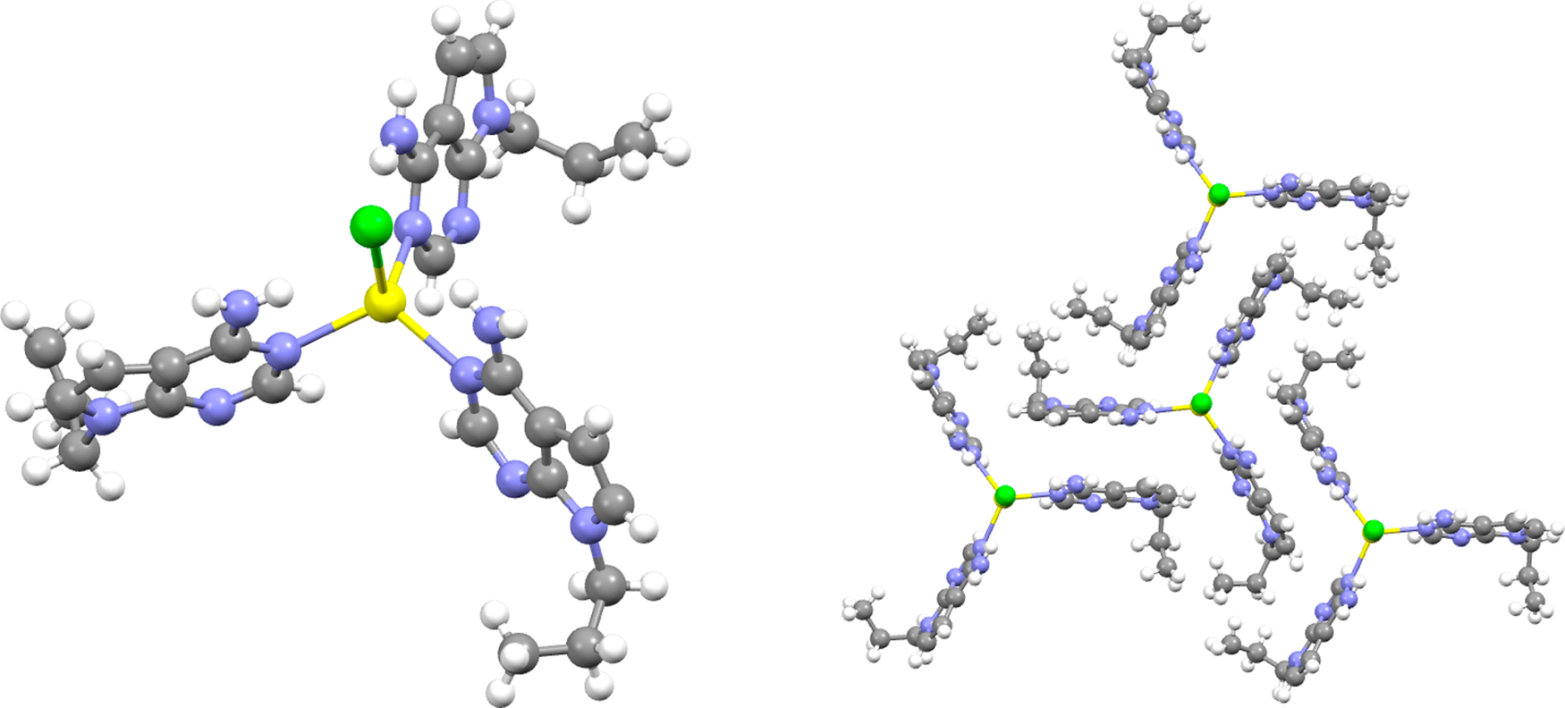
Left: Molecular structure of [Ag­(*N1-*pX)_3_Cl] (**6**). Right: Crystal packing diagram showing a 3D network resembling the Celtic Triskelion symbol. Color code: carbon, gray; nitrogen, blue; silver, yellow; chlorine, green; hydrogen, white.

To correlate these solid-state structural findings with solution behavior, ^1^H NMR spectroscopy was employed to study compounds **1** and **5**, which exhibit distinct coordination modes (monodentate via N1, or bidentate via N1 and N3 atoms, respectively). In both complexes, Ag^I^ coordination caused downfield shifts in the proton signals compared to the free pX base (Figure S5), in agreement with the NMR titration data for pX and Ag^I^ (Figure 5). Notably, when pX acts as a bidentate ligand through N1 and N3 atoms, as observed in complex **5**, the amino proton signals exhibit a more pronounced downfield shift. These observations are in line with our earlier titration results, further supporting the formation of a 1:1 stoichiometry between pX and Ag^I^ ions.

## Conclusions

This study answers a key question regarding the preferential formation of silver-modified DNA base pairs (X-Ag^I^-T), offering significant insights into the understanding of metalized 7-deaza-DNA structures. It was demonstrated that the X-Ag^I^-T heterobase pairs are preferentially formed over homobase pairs such as X-Ag^I^-X and T-Ag^I^-T, thus reinforcing structural stability and preserving the Watson–Crick organization within 7-deaza-DNA duplexes. CD spectroscopy, NMR spectroscopy, and ESI-MS confirmed the formation of Watson–Crick silver-modified **X**_**15**_ **·T**_**15**_ **-Ag**_**15**_ and complexes with a well-defined stoichiometry. Furthermore, the study also reveals the binding preferences of Ag^I^ ions in DNA sequences modified exclusively with 7-deazaadenine. A novel X-Ag^I^-X homobase pair was identified and characterized by means of NMR spectroscopy and X-ray diffraction, providing a new framework for designing silver-stabilized DNA architectures. More studies are currently underway to also evaluate the preferential formation of the counterpart 7-deazaguanine-Ag^I^-cytosine, which constitutes the other 7-deaza Watson–Crick silver-modified base pair.

## Experimental Section

### Coordination Compound Synthesis

Ligand pX was prepared as previously described.^28^ ^1^H NMR spectra registered for metal complexes were recorded with a Bruker AMX instrument working at 400 MHz. Elemental analyses were carried out with a Fisons-Carlo Erba analyzer model EA 1108. Infrared (IR) spectra were registered in a Bruker Tensor-27 FT-IR spectrometer.

#### Compound **1**, [Ag­(*N1*-pX)_2_]­(ClO_4_)

An aqueous solution of AgClO_4_ (0.017 g, 0.08 mmol) was added to an aqueous solution containing pX (0.03 g, 0.17 mmol) dropwise with stirring. The resulting mixture was stirred for 30 min and heated to 40 °C. The solution was left to crystallize by the evaporation of the solvent at room temperature. After a few days, single crystals appeared in solution suitable for X-ray crystallography. The crystals were filtered and dried in a vacuum. ^1^H NMR (400 MHz, DMSO-d_6_); δ 8.17 (s, 1H; CH), 7.29 (s, 2H; NH_2_), 7.23 (d, *J* = 3.3 Hz, 1H; CH), 6.60 (d, *J* = 3.3 Hz, 1H; CH), 4.09 (t, *J* = 7.0 Hz, 2H; CH_2_), 1.82–1.70 (m, 2H; CH_2_), 0.81 (t, *J* = 7.4 Hz, 3H; CH_3_). Elemental analysis corresponds to [C_18_H_24_N_8_ClO_4_Ag·(H_2_O)_1.3_]: calcd C 37.07, H 4.59,N 19.21; found: C 37.02, H 4.88, N 19.03. HRMS (ESI): *m*/*z* calcd for C_18_H_24_N_8_Ag [M + H]^+^; 459.1175; found, 459.1183. IR (cm^–1^): 1641 (s), 1598 (s), 1373 (w), 1257 (m), 1037 (s), 919 (w), 715 (s), 622 (s).

#### Compound **2**, [Ag­(*N1*-pX)_2_]­(NO_3_)

To a warm aqueous solution of AgNO_3_ (0.04 g, 0.25 mmol) was added an aqueous solution of pX (0.03 g, 0.17 mmol) dropwise under stirring. The clear solution was heated to 40 °C and left at this temperature with stirring for 30 min. The solution was left to crystallize by evaporation of the solvent at room temperature. After a few days, single crystals appeared in solution suitable for X-ray crystallography. The crystals were filtered and dried in vacuum. Elemental analysis corresponds to [C_18_H_24_N_8_NO_3_Ag]: calcd C 41.39, H 4.63,N 24.14; found: C 41.16, H 4.29, N 24.44. IR (cm^–1^): 1641 (m), 1554 (w), 1485­(w), 1311 (s), 1257 (s), 1000 (w), 713 (s), 601 (m).

#### Compound **3**, [Ag­(*N1*-pX)_2_]­(BF_4_)

To an aqueous solution of AgBF_4_ (0.03 g, 0.17 mmol) was added an aqueous solution containing pX (0.03 g, 0.17 mmol) dropwise with stirring. The solution thus obtained was heated to 40 °C and left at this temperature with stirring for 30 min. The solution was left to crystallize by evaporation of the solvent at room temperature. After a few days, single crystals appeared in solution suitable for X-ray crystallography. The crystals were filtered and dried in vacuum. Elemental analysis corresponds to [C_18_H_24_N_8_BF_4_Ag]: calcd C 39.52, H 4.42, N 20.48; found: C 39.16, H 4.69, N 20.09. IR (cm^–1^): 1641 (s), 1600 (s), 1488 (w), 1367 (m), 1375 (m), 1263 (m), 997 (s), 721 (s), 516 (w).

#### Compound **4**, [Ag­(*N1*-pX)_2_]­(CF_3_SO_3_)

To an aqueous solution of AgCF_3_SO_3_ (0.01 g, 0.038 mmol) was added an aqueous solution of pX (0.07 g, 0.099 mmol) dropwise with stirring. The resulting mixture was stirred for 30 min and covered by a plastic film to control the solvent evaporation. Then, the solution was left to crystallize by evaporation of the solvent at room temperature. After a few days, single crystals appeared in solution suitable for X-ray crystallography. The crystals were filtered and dried in vacuum. [(C_9_H_12_N_4_)_2_CF_3_SO_3_Ag·(H_2_O)_0.5_]: calcd C 36.9, H 4.08, N 18.12; found: C 37.31, H 4.36, N 18.64. IR (cm^–1^): 1645 (m), 1492 (m), 1367 (w), 1245 (s), 1163 (m), 1026 (m), 717 (m), 511 (w).

#### Compound **5**, [Ag_4_(*N1,N3*-pX)_4_(ClO_4_)_2_]­(ClO_4_)_2_

Ligand pX (44 mg, 0.25 mmol) was dissolved in a mixture of warm water and acetonitrile (2:1, 50 mL), and the solution was added dropwise to an aqueous solution of AgClO_4_ (52 mg, 0.25 mmol). The solution was stirred and filtered through a cellulose filter. The solution was left to crystallize, affording crystals suitable for X-ray crystallography. ^1^H NMR (400 MHz, DMSO-*d*_6_); δ 8.20 (s, 1H; CH), 7.45 (s, 2H; NH_2_), 7.25 (d, *J* = 3.5 Hz, 1H; CH), 6.63 (d, *J* = 3.5 Hz, 1H; CH), 4.1 (t, *J* = 7.0 Hz, 2H; CH_2_), 1.82–1.70 (m, 2H; CH_2_), 0.81 (t, *J* = 7.4 Hz, 3H; CH_3_). Elemental analysis corresponds to [C_9_H_12_N_4_ClO_4_Ag·(CH_3_CN)_0.25_]: calcd C 28.97, H 3.26, N 15.11; found: C 29.34, H 2.99, N 14.56.

#### Compound **6**, [Ag­(*N1*-pX)_3_]­(Cl)

The formation of this complex was unexpected and must be due to the presence of some chloride impurities during the synthesis and crystallization of compound **2**. These new crystals were analyzed by X-ray measurements. Unfortunately, there were not enough crystals to perform further characterization.

### Single-Crystal X-ray Diffraction Structure Determination

X-ray diffraction data for compounds **1–6** were collected on a Bruker D8 Venture diffractometer equipped with either a Cu (λ = 1.54178 Å) or a Mo (λ = 0.71073 Å) X-ray tube, a Bruker AXS Photon 100 detector, and a Kryoflex II cooling apparatus. X-ray diffraction data for compound **6** were collected using a Bruker X8 Proteum diffractometer equipped with a Cu (λ = 1.54178 Å) sealed rotating anode X-ray tube, a Bruker AXS Smart 6000 CCD detector, and an Oxford Cryostream 700 plus cooling apparatus. Data reduction was performed with the software APEX3,^29^ while data correction for absorption was carried out using the software SADABS.^30^ The structures were solved by the Patterson method and refined using least-squares minimization with the SHELX suite of programs^31–33^ integrated in OLEX2.^34^ The main crystallographic information and experimental and data treatment details are provided in Table S2.

### Oligonucleotide Synthesis

Oligonucleotide **X**_**15**_ was synthesized and characterized as reported previously.^35^ Oligonucleotides **A**_**15**_ and **T**_**15**_ were purchased from Integrated DNA Technologies, Inc. (IDT) with HPLC purification.

Molecular mass determinations for the **X**_**15**_ **·T**_**15**_ **-Ag**_**15**_ complex were performed by ESI-MS equipped with a time-of-flight analyzer (ESI-TOF MS) using a Micro Tof-Q Instrument (Bruker Daltonics GmbH, Bremen, Germany) calibrated with NaI (200 ppm of NaI in a 1:1 H_2_O: isopropanol mixture), interfaced with a Series 1100 HPLC pump (Agilent Technologies) equipped with an autosampler, both controlled by the Compass Software.

The hexanucleotide **XT** was synthesized on K&A Laborgeraete GbR DNA/RNA Synthesizer H-8 using standard phosphoramidite chemistry in the DMT-off mode, using phosphoramidites purchased from ChemBiotech (Germany), deprotected overnight in aqueous ammonia, and desalted on a Sephadex G25 column using a GE Akta Purifier. Analytical HPLC analysis was performed by RP-HPLC using a Thermo Scientific P4000 instrument in association with a Spectra System UV8000 detector and a Phenomenex Clarity 3 μ column (Figure S6), with gradient elution at 0.8 mL/min using Buffer A (0.1 M triethylammonium acetate, pH 6.5 + 5% CH_3_CN) and Buffer B (0.1 M triethylammonium acetate, pH 6.5 + 65% CH_3_CN). The mobile phase started at 100% A, shifted to 70% A/30% B at 20 min, then returned to 100% A at 21 min, and was held until 25 min for re-equilibration. The oligonucleotide was characterized by high-resolution ESI-MS using a QTOF system with ion mobility, BRUKER, model timsTOFPro2. Calcd. for XT [C_63_H_79_N_18_O_34_P_5_]: 1786.3 Da, found: 1786.2510 Da (Figure S7).

## Supplementary Material



## Notes

CD spectra for the titration of the reference duplex **A**_**15**_·**T**_**15**_, UV-melting curves for **X**_**15**_, NMR spectra for **(XT)**_**6**_ upon adding Ag^I^ ions, as well as for complexes **1** and **5**, table for crystallographic data and structure refinement details of compounds **1–6**, and tables for weak π-noncovalent interactions present in the crystal structures (PDF)
