# Supplementary material for: Silver Binding Dichotomy for 7‑Deazaadenine/Thymine: Preference for Watson–Crick Pairing over Homobase Interactions in DNA
Source: Inorg Chem. 2025 Jul 10;64(28):14455–65. doi: 10.1021/acs.inorgchem.5c01762 (PMC12284849; doi:10.1021/acs.inorgchem.5c01762)
Supplement: Supplementary file 1 [file ic5c01762_si_001.pdf]

Supporting Information

Silver Binding Dichotomy for 7-Deazaadenine/Thymine: Preference for Watson-Crick Pairing Over Homobase Interactions in DNA

Carmen López-Chamorro [a], Antonio Pérez-Romero [a], Alicia Domínguez-Martín [a], Uroš Javornik [b], Oscar Palacios[c], Janez Plavec [b], Miguel A. Galindo [a]*

[a] Departamento de Química Inorgánica, Universidad de Granada Avda. Fuentenueva s/n, 18071 Granada, Spain.

[b] Slovenian NMR Center, National Institute of Chemistry and Faculty of Chemistry and Chemical Technology, University of Ljubljana, SI-1000 Ljubljana, Slovenia

[c] Departament de Química, Facultat de Ciències, Universitat Autònoma de Barcelona, Cerdanyola del Vallès 08193, Spain.

e-mail: magalindo@ugr.es

Content

Circular dichroism of Duplex $A_{15}{\cdot}T_{15}$ upon adding Ag^{I} ions 3

UV melting curves for X_{15} upon adding Ag^{I} ions 4

NMR spectroscopy titration of duplex $(XT)_6$ with Ag^{I} ions 4

NMR spectra for Complex 1 and Complex 5 5

Oligonucleotide Mass spectrometry and HPLC 6

Single-Crystal X-ray Crystallographic data 7

Weak π-non-covalent interactions present in the crystal structure of Compound 1, 2 and 3 8

 Compound 3 - $[Ag(N1\text{-}pX)_2]BF_4$ 8

 Compound 1 - $[Ag(N1\text{-}pX)_2]ClO_4$ 9

 Compound 2 - $[Ag(N1\text{-}pX)_2]NO_3$ 9

Weak π-non-covalent interactions present in the crystal structure of Compound 4 11

 Compound 4 - $[Ag(N1\text{-}pX)_2]\ CF_3SO_3$ 11

Weak non-covalent interactions present in the crystal structure of Compound 5 14

 Compound 5 - $[Ag_4(\textit{N1,N3}\text{-}pX)_4(ClO_4)_2](ClO_4)_2$ 16

Circular dichroism of Duplex $A_{15}·T_{15}$ upon adding Ag^{I} ions

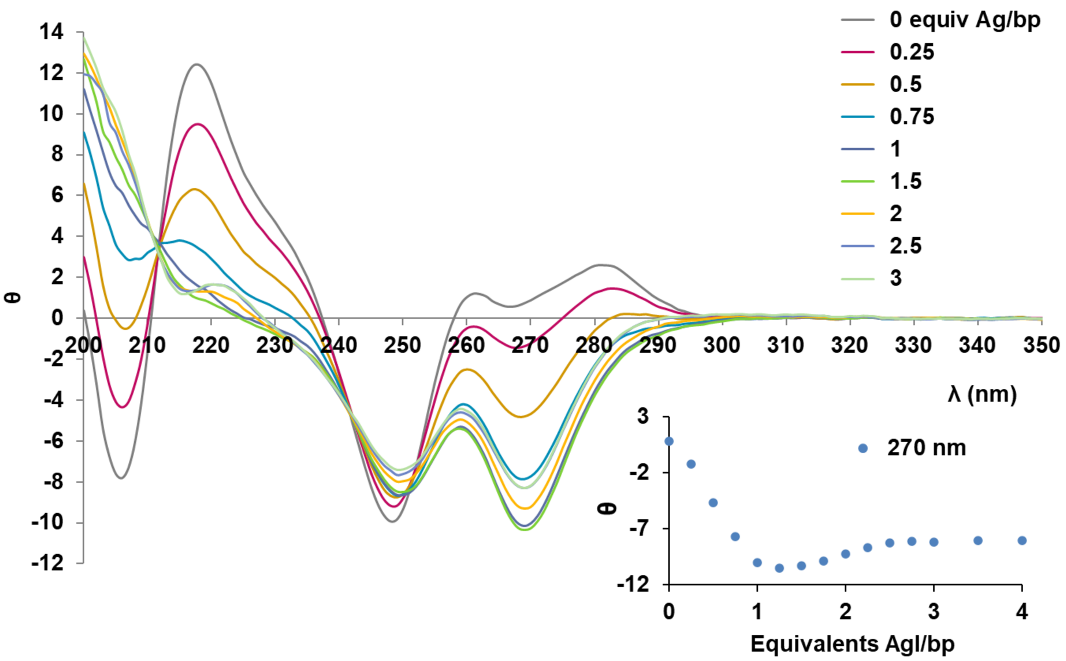

Figure S1. CD spectra of duplex **$A_{15}·T_{15}$** upon addition of different amounts of Ag^{I} ions per base pair (bp). Insets: changes in the CD at 270nm. Experimental conditions: 2 µM of duplex, 100 mM $NaClO_4$ and 5 mM MOPS buffer pH 6.8-7.

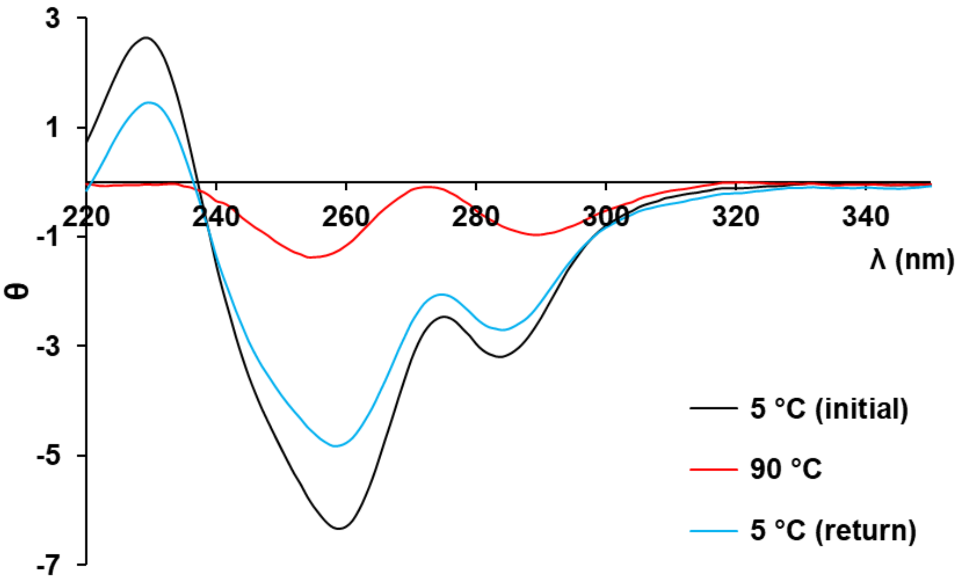

Figure S2. CD spectra of duplex **$X_{15}·T_{15}$** + 1 equiv. Ag^{I} ions per base pair (bp) at different temperatures. The sample was initially recorded at 5 °C (initial), then heated to 90 °C, and finally cooled back to 5 °C (return). Experimental conditions: 2 µM of duplex, 100 mM $NaClO_4$ and 5 mM MOPS buffer pH 6.8-7. Note: Variations in the intensity of the spectra recorded at 5 °C are attributed to evaporation during the heating process, as the cuvette was covered with a cap but not fully sealed.

UV melting curves for X_{15} upon adding Ag^{I} ions

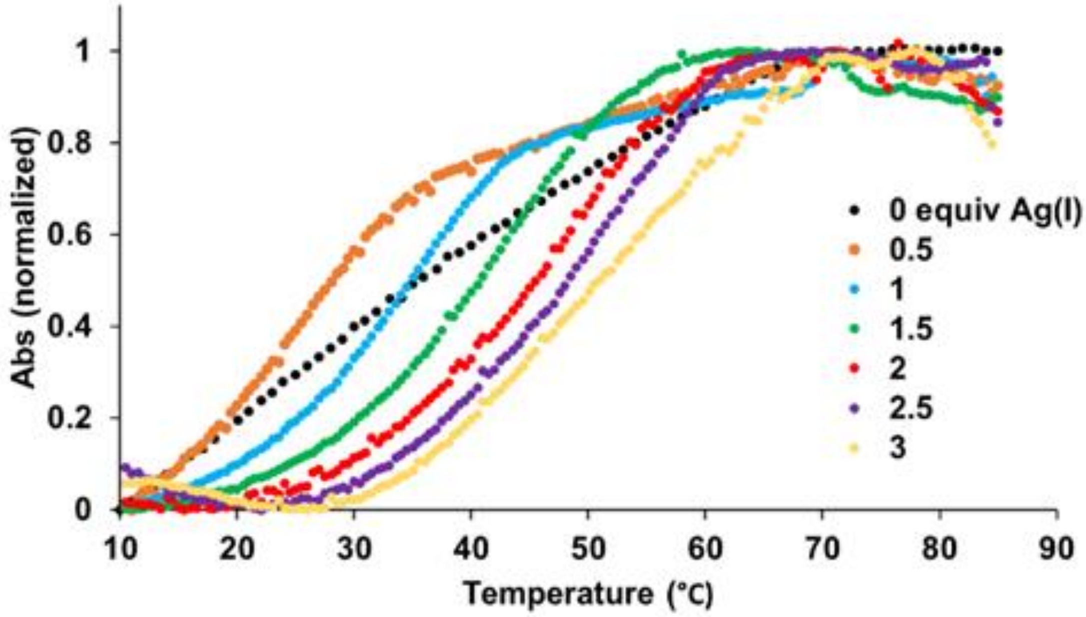

Figure S3. Melting curves for $\mathbf{X_{15}}$ in absence and presence of controlled amount of $AgNO_3$. Conditions: 2 µM of ds-oligonucleotide, 100 mM $NaClO_4$ and 5 mM MOPS buffer at pH 6.8-7, $AgNO_3$ 0-45µM. Absorbance measured at 260nm.

NMR spectroscopy titration of duplex $(XT)_6$ with Ag^{I} ions

The NMR sample to study the interaction of duplex $\mathbf{(XT)_6}$ and Ag(I) ions contained 0.22 mM DNA in 100 mM Tris/HNO_3 buffer with pH 8.6, 100 mM $NaClO_4$, and 10 % (v/v) D_2O. Spectra were recorded on an Agilent Technologies DD2 600 MHz NMR spectrometer with a HCN cold probe at 5 and 25 °C. The sample was titrated with an aqueous solution of $AgNO_3$ up to 6 equivalents Ag^{I} per base pair (3.96 mM $AgNO_3$). ^{1}H-NMR spectra of the titration points were recorded at 5 °C using excitation sculpting for water suppression, 16384 points, and 1.5 s relaxation delay.

DOSY spectra were recorded on DNA samples without and with 6 equivalents Ag/bp. The samples were freeze dried and redissolved in D_2O. The spectra were recorded at 25 °C using the DgcsteSL_cc pulse sequence, with 32768 points, 1.0 s relaxation delay, 4 ms gradient time (little delta), and 60 ms diffusion delay time (big delta). 12 increments were recorded for each experiment with gradient strengths between 0 and 60 gauss/cm. Additionally NOESY, TOCSY and DQF-COSY spectra were recorded at the start and end points of the titration to facilitate chemical shift assignments. Chemical shifts were referenced externally to DSS in water.

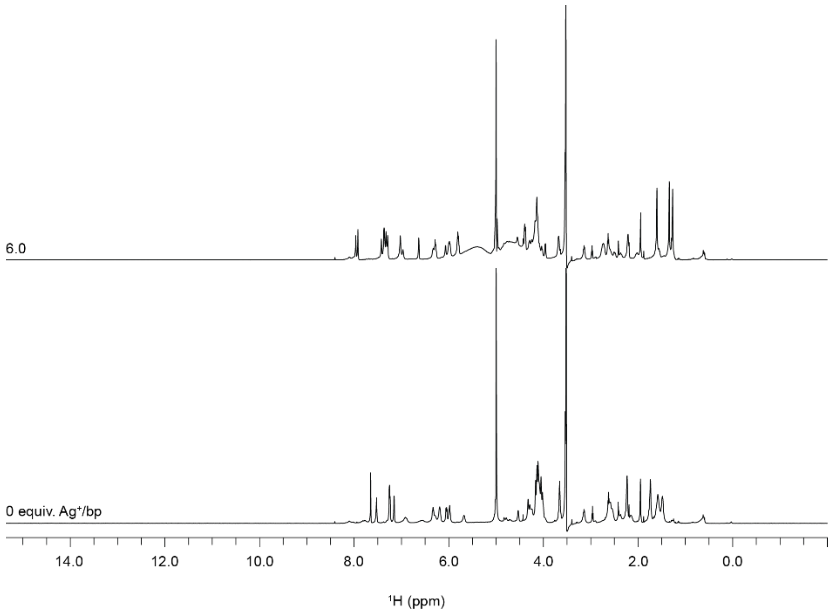

Figure S4. ^{1}H NMR spectra of duplex **(XT)$_6$** at 0 and 6 equivalents Ag^{I}/base pir. The sample contained 0.22 mM DNA in 100 mM Tris/HNO_3 buffer, pH 8.6, with 100 mM $NaClO_4$. The spectra were recorded at 5 °C.

NMR spectra for Complex 1 and Complex 5

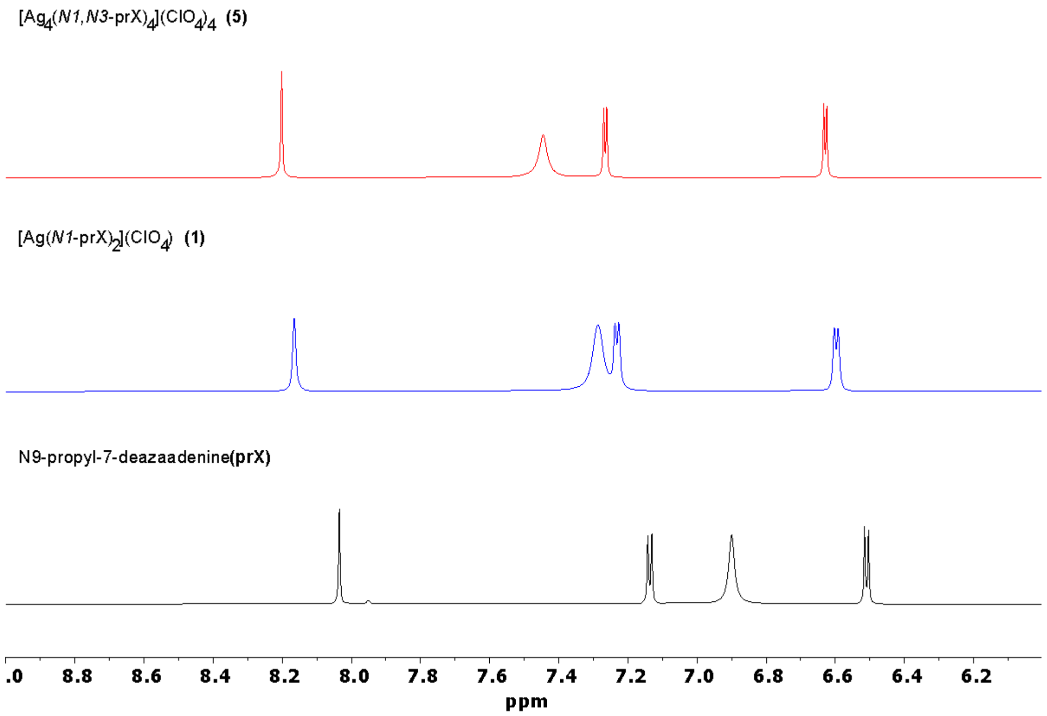

Figure S5. Aromatic region of ^{1}H-NMR (DMSO-d_6) spectra for free N9-propyl-7-deazaadenine base (pX) and complexes [Ag(*N1*-pX)$_2$](ClO_4) (**1**) and [Ag$_4$(*N1,N3*-pX)$_4$(ClO_4)$_2$](ClO_4)$_2$ (**5**).

Oligonucleotide Mass spectrometry and HPLC

Table S1. Experimental and theoretical masses of the species formed for $\mathbf{X_{15} \cdot T_{15}}$ in the presence of $AgNO_3$.

	14 Ag	15 Ag
**Exp. Mass**	10615.8	10723.0
**Theor. Mass**	10616.5	10723.4

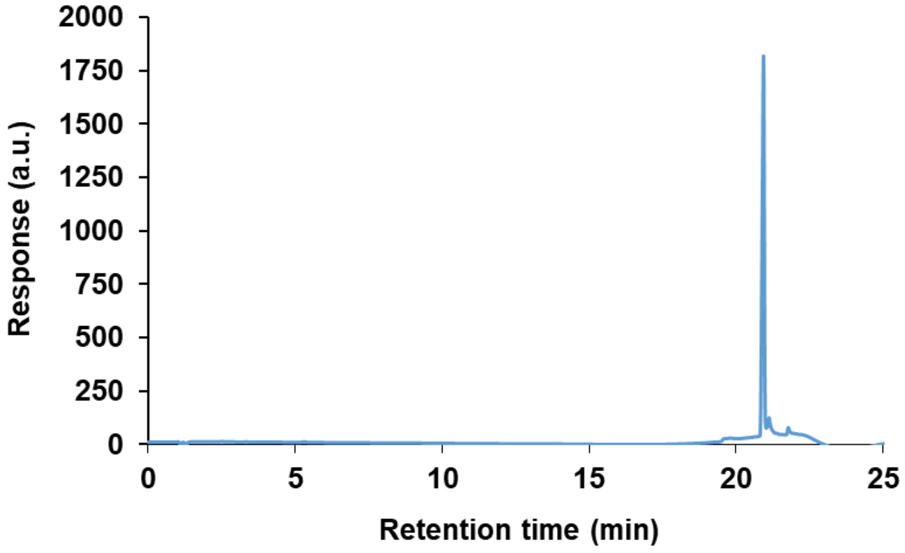

Figure S6. HPLC trace for oligonucleotide **XT**.

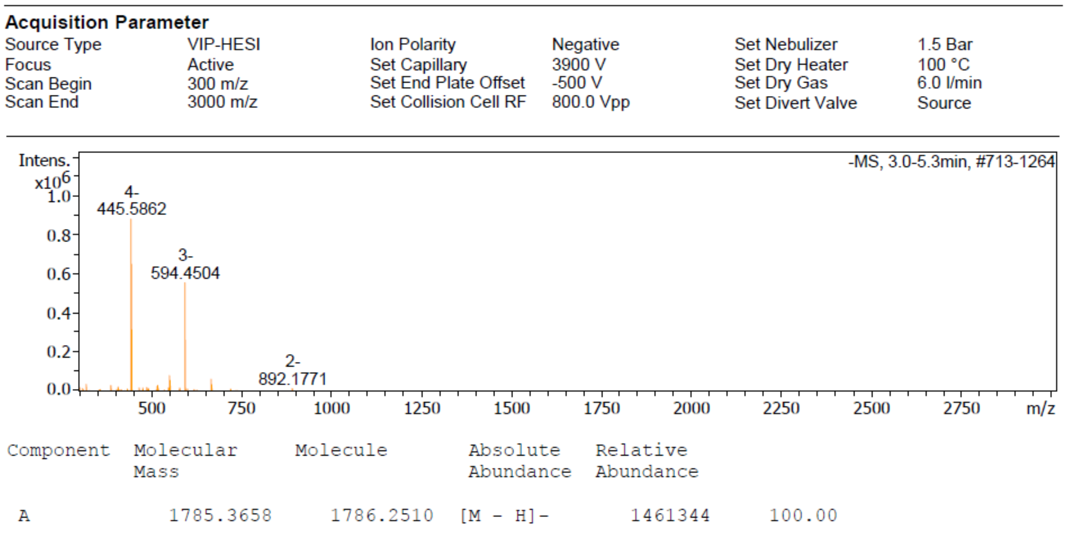

Figure S7. ESI-MS (neg. mode) spectrum and deconvolution report for oligonucleotide **XT**.

Single-Crystal X-ray Crystallographic data

Table S2. Crystallographic data and structure refinement details of compounds **1-6**

**Compound Name**	**1**	**2**	**3**	**4**	**5**	**6**
Temperature/K	100(2)	105(2)	105(2)	100(2)	100(2)	100(2)
Formula	$C_{18}H_{26}AgClN_8O_5$	$C_{18}H_{24}AgN_9O_3$	$C_{18}H_{24.60}AgBF_4N_8O_{0.3}$	$C_{19}H_{29}AgF_3N_8O_{5.50}S$	$C_{36}H_{48}Ag_4Cl_4N_{16}O_{16}$	$C_{27}H_{36}AgClN_{12}$
Formula Weight	577.79	522.33	552.54	654.43	1534.18	672.00
Crystal system	Monoclinic	Monoclinic	Monoclinic	Triclinic	Triclinic	Hexagonal
Space group	$P\ 2_1/n$	$P\ 2_1/c$	$P\ 2_1/c$	P-1	P-1	P 6c
a/Å	16.1583(12)	15.5989(8)	15.7746(10)	9.7541(4)	9.2261(4)	15.4163(13)
b/Å	9.6926(8)	9.4363(5)	9.7554(6)	13.9080(6)	15.8333(8)	15.4163(13)
c/Å	16.6795(13)	16.2993(8)	16.5821(9)	20.0815(7)	18.3459(9)	7.1248(7)
α/°	90	90	90	81.5230(10)	68.714(2)	90
β/°	117.922(3)	115.057(2)	117.686(2)	82.1430(10)	79.691(2)	90
γ/°	90	90	90	78.6810(10)	77.351(2)	120
V/Å^3	2308.2(3)	2173.4(2)	2259.6(2)	2625.72(18)	2421.9(2)	1466.4(3)
Z	4	4	4	4	2	2
D_c/g cm^{-3}	1.663	1.596	1.624	1.655	2.104	1.522
μ/mm^{-1}	1.036	0.967	0.949	0.917	1.902	0.819
F(000)	1176	1064	1116	1332	1520	692
θ range/°	2.406 – 30.510	2.514 – 28.333	2.507 – 28.360	1.927 – 28.285	2.276 – 23.315	2.642 – 27.503
Reflections collected	57004	21782	48272	98160	55134	11116
Unique reflections	7039	5363	5602	12921	7015	2240
R_{int}	0.0502	0.0486	0.0271	0.0523	0.0297	0.0602
Data/restraints/parameters	7039/0/303	5363/0/282	5602/0/303	12921/0/695	7015/4/686	2240/1/128
Goodness of fit (F^2)	1.068	1.489	1.066	1.162	1.105	1.096
$R1$ (I > 2σ (I))	0.0314	0.0746	0.0230	0.0454	0.0327	0.0378
$wR2$ (I > 2σ (I))	0.0816	0.1880	0.0608	0.1185	0.0739	0.0782

Weak π-non-covalent interactions present in the crystal structure of Compound 1, 2 and 3

==
==========
Analysis of Short Ring-Interactions with Cg-Cg Distances < 6.0 Ang., Alpha < 20.000 Deg. and Beta < 60.0 Deg.
==
==========

- Cg(I) = Plane number
- Alpha = Dihedral Angle between Planes I and J (Deg)
- Beta = Angle Cg(I)-->Cg(J) or Cg(I)-->Me vector and normal to plane I (Deg)
- Gamma = Angle Cg(I)-->Cg(J) vector and normal to plane J (Deg)
- Cg-Cg = Distance between ring Centroids (Ang.)
- CgI_Perp = Perpendicular distance of Cg(I) on ring J (Ang.)
- CgJ_Perp = Perpendicular distance of Cg(J) on ring I (Ang.)
- Slippage = Distance between Cg(I) and Perpendicular Projection of Cg(J) on Ring I (Ang).
- P,Q,R,S = J-Plane Parameters for Carth. Coord. (Xo, Yo, Zo)

Compound 3 - [Ag(N1-pX)$_2$]BF_4

Cg(I) Res(I)	Cg(J) [ARU(J)]	Cg-Cg	Transformed J-Plane P, Q, R, S	Alpha	Beta	Gamma	CgI_Perp	CgJ_Perp	Slippage
Cg3 [1] ->	Cg3 [3666.01]	3.8689(10)	-0.6569 0.6136-0.4382 -4.6012	0.03(7)	26.8	26.8	3.4533(6)	3.4533(6)	1.744

[3666] = 1-X,1-Y,1-Z

6-Membered Ring (Cg3) N1A --> C2A --> N3A --> C4A --> C5A --> C6A

Equivalent, although much weaker interactions are found in compounds 1 and 2:

Compound 1 - $[Ag(N1\text{-}pX)_2]ClO_4$

Cg(I) Res(I) Cg(J) [ARU(J)] Cg-Cg Transformed J-Plane P, Q, R, S Alpha Beta Gamma CgI_Perp CgJ_Perp Slippage

Cg4 [1] -> Cg4 [3655.01] 4.0351(12) -0.6191-0.6199 0.4821 -3.2639 0.00(9) 30.5 30.5 3.4762(8) 3.4762(8) 2.049

[3655] = 1-X,-Y,-Z

6-Membered Ring (Cg4) N1B --> C2B --> N3B --> C4B --> C5B --> C6B

Compound 2 - $[Ag(N1\text{-}pX)_2]NO_3$

Cg(I) Res(I) Cg(J) [ARU(J)] Cg-Cg Transformed J-Plane P, Q, R, S Alpha Beta Gamma CgI_Perp CgJ_Perp Slippage

Cg4 [1] -> Cg4 [3656.01] 3.902(4) -0.6564-0.6308-0.4137 -4.2215 0.0(3) 30.2 30.2 3.374(3) 3.374(3) 1.962

[3656] = 1-X,-Y,1-Z

6-Membered Ring (Cg4) N1B --> C2B --> N3B --> C4B --> C5B --> C6B

==

Ring-Metal Interactions with Cg-Me < 4.0 Ang.

==

Cg(I) Res(I) Me(J) [ARU(J)] Cg(I)-Me(J) MeJ_Perp Beta

Cg1 [1] -> Ag1 [3666.01] 3.466 3.097 26.67

[3666] = 1-X,1-Y,1-Z

5-Membered Ring (Cg1) N9A --> C4A --> C5A --> C7A --> C8A

==
==========

Analysis of C-H...Cg(Pi-Ring) Interactions (H..Cg < 3.0 Ang. - Gamma < 40.0 Deg)

==
==========

- Cg(J) = Center of gravity of ring J (Plane number above)
- H-Perp = Perpendicular distance of H to ring plane J
- Gamma = Angle between Cg-H vector and ring J normal
- X-H..Cg = X-H-Cg angle (degrees)
- X..Cg = Distance of X to Cg (Angstrom)
- X-H, Pi = Angle of the X-H bond with the Pi-plane (i.e. Perpendicular = 90 degrees, Parallel = 0 degrees)

X--H(I)	Res(I)	Cg(J)	[ARU(J)]	H..Cg	Transformed J-Plane P, Q, R, S	H-Perp	Gamma	X-H..Cg	X..Cg	X-H,Pi
C10B -H02B	[1]	-> Cg1	[3676.01]	2.91	-0.6852 0.5855-0.4332 0.8480	-2.88	8.49	124	3.561(2)	37
C12B -H23B	[1]	-> Cg4	[2756.01]	2.94	0.6467 0.6648 0.3739 23.7807	-2.67	24.61	112	3.432(3)	46

[3676] = 1-X,2-Y,1-Z

[2756] = 2-X,1/2+Y,3/2-Z

5-Membered Ring (Cg1) N9A --> C4A --> C5A --> C7A --> C8A

6-Membered Ring (Cg4) N1B --> C2B --> N3B --> C4B --> C5B --> C6B

Weak π-non-covalent interactions present in the crystal structure of Compound 4

==
==========

Analysis of Short Ring-Interactions with Cg-Cg Distances <   6.0 Ang., Alpha < 20.000 Deg. and Beta < 60.0 Deg.

==
==========

- Cg(I)   = Plane number
- Alpha   = Dihedral Angle between Planes I and J (Deg)
- Beta   = Angle Cg(I)-->Cg(J) or Cg(I)-->Me vector and normal to plane I (Deg)
- Gamma   = Angle Cg(I)-->Cg(J) vector and normal to plane J (Deg)
- Cg-Cg   = Distance between ring Centroids (Ang.)
- CgI_Perp = Perpendicular distance of Cg(I) on ring J (Ang.)
- CgJ_Perp = Perpendicular distance of Cg(J) on ring I (Ang.)
- Slippage = Distance between Cg(I) and Perpendicular Projection of Cg(J) on Ring I (Ang).
- P,Q,R,S = J-Plane Parameters for Carth. Coord. (Xo, Yo, Zo)

Compound 4 - [Ag(N1-pX)$_2$] CF_3SO_3

Cg(I) Res(I)   Cg(J) [ARU(J)]   Cg-Cg   Transformed J-Plane P, Q, R, S   Alpha Beta Gamma   CgI_Perp   CgJ_Perp Slippage

Cg1 [1] -> Cg2 [2666.01] 3.7700(15) 0.6628-0.7402 0.1130 -1.5230 5.19(14) 26.0 20.9 3.5226(10) 3.3886(11)
1.652

Cg2 [1] -> Cg3 [2666.01] 3.5623(14) 0.6257-0.7609 0.1719 -1.0607 4.16(13) 17.7 17.5 3.3975(11) 3.3945(9)
1.081

Cg7 [2] -> Cg8 [2765.02] 3.7038(16) 0.6275-0.7771 0.0484 3.1277 2.58(15) 21.2 23.7 3.3921(11)
3.4537(11) 1.338

Cg7 [2] -> Cg10 [2765.02] 3.4897(14) 0.6337-0.7715 0.0564 3.2569 2.24(13) 15.4 13.8 3.3890(11)
3.3644(9) 0.927

[2666] = 1-X,1-Y,1-Z; [2765] = 2-X,1-Y,-Z

5-Membered Ring (Cg1) N9A --> C4A --> C5A --> C7A --> C8A

5-Membered Ring (Cg2) N9B --> C4B --> C5B --> C7B --> C8B

6-Membered Ring (Cg3) N1A --> C2A --> N3A --> C4A --> C5A --> C6A

5-Membered Ring (Cg7) N9C --> C4C --> C5C --> C7C --> C8C

5-Membered Ring (Cg8) N9D --> C4D --> C5D --> C7D --> C8D

6-Membered Ring (Cg10) N1D --> C2D --> N3D --> C4D --> C5D --> C6D

==
Ring-Metal Interactions with Cg-Me < 4.0 Ang.
==

Cg(I) Res(I) Me(J) [ARU(J)] Cg(I)-Me(J) MeJ_Perp Beta

Cg1	[1] -> Ag1	[2766.01]	3.702	-3.057	34.33
Cg8	[2] -> Ag2	[2665.02]	3.682	3.004	35.32

[2766] = 2-X,1-Y,1-Z ; [2665] = 1-X,1-Y,-Z

5-Membered Ring (Cg1) N9A --> C4A --> C5A --> C7A --> C8A

5-Membered Ring (Cg8) N9D --> C4D --> C5D --> C7D --> C8D

==

Analysis of C-H...Cg(Pi-Ring) Interactions (H..Cg < 3.0 Ang. - Gamma < 40.0 Deg)

==

- Cg(J) = Center of gravity of ring J (Plane number above)
- H-Perp = Perpendicular distance of H to ring plane J
- Gamma = Angle between Cg-H vector and ring J normal
- X-H..Cg = X-H-Cg angle (degrees)
- X..Cg = Distance of X to Cg (Angstrom)
- X-H, Pi = Angle of the X-H bond with the Pi-plane (i.e. Perpendicular = 90 degrees, Parallel = 0 degrees)

X--H(I) Res(I) Cg(J) [ARU(J)] H..Cg Transformed J-Plane P, Q, R, S H-Perp Gamma X-H..Cg X..Cg X-H,Pi

C12B -H23B [1] -> Cg4 [2656.01] 2.73 0.6801-0.7231 0.1206 6.9099 -2.67 12.22 140 3.539(3) 62

[2656] = 1-X,-Y,1-Z

6-Membered Ring (Cg4) N1B --> C2B --> N3B --> C4B --> C5B --> C6B

Weak non-covalent interactions present in the crystal structure of Compound 5

==

Analysis of Potential Hydrogen Bonds and Schemes with d(D...A) < R(D)+R(A)+0.50, d(H...A) < R(H)+R(A)-0.12 Ang., D-H...A > 100.0 Deg

==

Nr	Donor -- H···Acceptor	[ARU]	D - H	H...A	D...A	D -H...A
1	1 N6A --H61A ..O12	[2665.03]	0.88	2.04	2.832(6)	149
2	1 N6B --H61B ..O5	[2566.02]	0.88	2.18	2.988(6)	152
3	1 N6C --H61C ..O5	[2566.02]	0.88	2.16	2.906(7)	142
4	1 N6D --H61D ..O12	[2665.03]	0.88	2.03	2.879(6)	161
5	1 N6A --H62A ..O13	[2665.01]	0.88	2.26	3.100(5)	160
6	1 N6A --H62A ..O15	[2665.01]	0.88	2.47	3.168(5)	136'
7	1 N6B --H62B ..O9	[2666.03]	0.88	2.24	3.078(6)	159

8	1 N6B	--H62B	..O10	[2666.03]	0.88	2.48	3.159(5)	134'
9	1 N6C	--H62C	..O3	[2566.01]	0.88	2.05	2.876(5)	157
10	1 N6D	--H62D	..O8	[2565.02]	0.88	2.20	3.078(6)	173
11	1 C10A	--H01A	..O8	[1655.02]	0.99	2.54	3.525(7)	171
12	1 C2B	--H2B	..O7	[1555.02]	0.95	2.37	3.244(7)	153
13	1 C2C	--H2C	..O6	[1555.02]	0.95	2.45	3.246(6)	141
14	1 C2D	--H2D	..O6	[1555.02]	0.95	2.34	3.099(7)	136
15	1 C7B	--H7B	..O9	[2666.03]	0.95	2.52	3.308(6)	140
16	1 C8A	--H8A	..O1	[2675.01]	0.95	2.52	3.469(6)	173
17	1 C10D	--H01D	..O15	[1455.01]	0.99	2.49	3.076(7)	118
18 Intra	1*C11'	--H11'	..O4	[ ]	0.99	2.57	3.318(10)	132
19	1 C11D	--H12D	..O1	[1545.01]	0.99	2.56	3.358(7)	138

[2665.] = [2_665] = 1-x,1-y,-z ; [2566.] = [2_566] = -x,1-y,1-z ; [2675.] = [2_675] = 1-x,2-y,-z ; [2565.] = [2_565] = -x,1-y,-z

[1455.] = [1_455] = -1+x,y,z ; [1545.] = [1_545] = x,-1+y,z ; [2666.] = [2_666] = 1-x,1-y,1-z ; [1655.] = [1_655] = 1+x,y,z

For C--H...Acceptor Interactions See: Th. Steiner, Cryst. Rev, (1996), 6, 1-57

H-Bond classification [G.A.Jeffrey, H.Maluszynska & J.Mitra., Int.J.Biol.Macromol.(1985),7,336-348]

==
==========

Analysis of Short Ring-Interactions with Cg-Cg Distances < 6.0 Ang., Alpha < 20.000 Deg. and Beta < 60.0 Deg.

==
==========

- Cg(I) = Plane number
- Alpha = Dihedral Angle between Planes I and J (Deg)
- Beta = Angle Cg(I)-->Cg(J) or Cg(I)-->Me vector and normal to plane I (Deg)
- Gamma = Angle Cg(I)-->Cg(J) vector and normal to plane J (Deg)
- Cg-Cg = Distance between ring Centroids (Ang.)
- CgI_Perp = Perpendicular distance of Cg(I) on ring J (Ang.)
- CgJ_Perp = Perpendicular distance of Cg(J) on ring I (Ang.)
- Slippage = Distance between Cg(I) and Perpendicular Projection of Cg(J) on Ring I (Ang).
- P,Q,R,S = J-Plane Parameters for Carth. Coord. (Xo, Yo, Zo)

Compound 5 - $[Ag_4(\textit{N1,N3}\text{-pX})_4(ClO_4)_2](ClO_4)_2$

Cg(I)	Res(I)	Cg(J)	[ARU(J)]	Cg-Cg	Transformed J-Plane P, Q, R, S	Alpha	Beta	Gamma	CgI_Perp	CgJ_Perp	Slippage
Cg1	[1] ->	Cg8	[2665.01]	3.597(3)	0.6777-0.7135 0.1777 0.2835	3.3(3)	21.9	20.4	3.371(2)	3.3369(19)	1.343
Cg2	[1] ->	Cg3	[2566.01]	3.579(3)	0.6881-0.7245 0.0395 -6.9657	5.2(3)	15.6	12.5	3.494(2)	3.448(2)	0.961
Cg3	[1] ->	Cg6	[2566.01]	3.799(3)	0.6490-0.7606 0.0159 -7.2181	3.3(3)	28.6	25.3	3.435(2)	3.336(2)	1.818

Cg6 [1] -> Cg7 [2566.01] 3.778(3) -0.7163 0.6971-0.0303 6.4853 5.4(2) 28.7 28.5 3.319(2) 3.313(2) 1.816

[2665] = 1-X,1-Y,-Z: [2566] = -X,1-Y,1-Z

5-Membered Ring (Cg1) N9A --> C4A --> C5A --> C7A --> C8A

5-Membered Ring (Cg2) N9B --> C4B --> C5B --> C7B --> C8B

5-Membered Ring (Cg3) N9C --> C4C --> C5C --> C7C --> C8C

6-Membered Ring (Cg6) N1B --> C2B --> N3B --> C4B --> C5B --> C6B

6-Membered Ring (Cg7) N1C --> C2C --> N3C --> C4C --> C5C --> C6C

6-Membered Ring (Cg8) N1D --> C2D --> N3D --> C4D --> C5D --> C6D

==

Ring-Metal Interactions with Cg-Me < 4.0 Ang.

Cg(I) Res(I)	Me(J)	[ARU(J)]	Cg(I)-Me(J)	MeJ_Perp	Beta
Cg2 [1]	-> Ag3	[2666.01]	3.434	-2.961	30.43
Cg4 [1]	-> Ag1	[2565.01]	3.340	-3.217	15.56
Cg5 [1]	-> Ag1	[2665.01]	3.847	3.063	37.23
Cg8 [1]	-> Ag1	[2565.01]	3.630	3.204	28.05

[2666] = 1-X,1-Y,1-Z

[2565] = -X,1-Y,-Z

[2665] = 1-X,1-Y,Z

5-Membered Ring (Cg2) N9B --> C4B --> C5B --> C7B --> C8B

5-Membered Ring (Cg4) N9D --> C4D --> C5D --> C7D --> C8D

6-Membered Ring (Cg5) N1A --> C2A --> N3A --> C4A --> C5A --> C6A

6-Membered Ring (Cg8) N1D --> C2D --> N3D --> C4D --> C5D --> C6D
